# Unexpected Large Photosynthetic Thermal Plasticity of Montane Andean Trees

**DOI:** 10.1111/gcb.70266

**Published:** 2025-05-23

**Authors:** Mirindi Eric Dusenge, Sebastian González‐Caro, Zorayda Restrepo, Anna Gardner, Patrick Meir, Iain P. Hartley, Stephen Sitch, Adriana Sanchez, Juan Camilo Villegas, Lina M. Mercado

**Affiliations:** ^1^ Division of Plant Sciences, Research School of Biology The Australian National University Canberra Australian Capital Territory Australia; ^2^ Faculty of Environment, Science, and Economy University of Exeter Exeter UK; ^3^ Grupo de Servicios Ecosistémicos y Cambio Climático Corporación COL‐TREE Medellín Colombia; ^4^ Birmingham Institute of Forest Research University of Birmingham Birmingham UK; ^5^ School of Geosciences University of Edinburgh Edinburgh UK; ^6^ Departamento de Biología, Facultad de Ciencias Naturales Universidad del Rosario Bogotá Colombia; ^7^ Grupo en Ecología Aplicada, Escuela Ambiental, Facultad de Ingeniería Universidad de Antioquia Medellín Colombia; ^8^ UK Centre for Ecology & Hydrology Wallingford UK

**Keywords:** acclimation, climate change, photosynthesis, tropical montane forests, *V*_cmax_ and *J*_max_

## Abstract

Tropical forests play a significant role in global carbon sequestration. However, our understanding of how tropical tree species adjust to climate warming remains limited to studies on seedlings grown in pots and highly controlled growth conditions. To reduce this knowledge gap, we used a field experiment with 5‐year‐old juvenile trees of 12 naturally co‐occurring dominant tropical Andean montane and lowland species growing in three common gardens established along a natural thermosequence in the tropical Andes. Based on a few previous studies, we hypothesized that montane species would exhibit a weaker photosynthetic thermal acclimation capacity compared to lowland counterparts. Our results showed that montane tree species can thermally acclimate net photosynthesis by shifting their thermal optimum (*T*_opt_) by 0.6°C per 1°C of warming. This strong shift in *T*_opt_ was correlated to simultaneous strong shifts in *T*_opt_ of apparent photosynthetic capacity parameters (*V*_cmax_ and *J*_max_), which increased by 0.7°C per 1°C of warming. This strong thermal acclimation resulted in similar rates of net CO_2_ assimilation between montane and lowland species across different thermal environments. At last, rates of net photosynthesis at growth temperature explained 30% of the variation in the relative tree growth rates across the two species groups and thermal environments. Our results suggest that the strong physiological acclimation of photosynthesis to warming among montane Andean tree species should be considered when predicting future impacts of warming on Andean plant communities.

## Graphical abstract

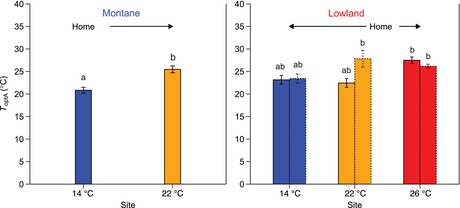

## 1 Introduction

Tropical forests are important globally as they store the largest amount of carbon in aboveground biomass (Pan et al. 2011; Xu et al. 2021). Among these tropical forests are tropical montane forests (TMFs; > 1000 m above sea level; Spracklen and Righelato 2014) which comprise only about 8.3% of the total tropical forest area (Spracklen and Righelato 2014). Despite their relatively small coverage, these forests are remarkable for their rich species diversity and endemism (Kessler and Kluge 2008; Myers et al. 2000), as well as their capacity to store significant amounts of carbon in live biomass (Cuni‐Sanchez et al. 2021; Duque et al. 2021; Nyirambangutse et al. 2017). Among these TMFs are the Andean forests, which constitute the most extended continental mountain range in the world, spanning seven countries: Venezuela, Colombia, Ecuador, Peru, Bolivia, Chile, and Argentina (Aide et al. 2019; Pérez‐Escobar et al. 2022). Andean forests are critical global biodiversity hotspots (Myers et al. 2000) and have relatively higher annual carbon sink strength per hectare than lowland Amazonian counterparts, despite their relatively lower aboveground carbon stocks (Duque et al. 2021). However, these tropical montane ecosystems are experiencing the fastest warming rate compared to lowland ecosystems (Mata‐Guel et al. 2023). There is growing empirical evidence that climate warming in the Andean region (Russell et al. 2017) is altering the species composition of the Andes forest communities (Feeley, Bravo‐Avila, et al. 2020). This shift has led to a relative increase in the abundance of lowland species at higher‐elevation sites, which were traditionally inhabited by montane species, in a phenomenon known as thermophilization (Duque et al. 2015; Fadrique et al. 2018). The concomitant decrease in montane species was recently shown to be linked to mortality (Duque et al. 2015), although the exact mechanisms driving this mortality are not well known. This thermophilization is also leading to changes in plant functional traits, such as the increase in species with high dry matter content and low leaf area (Aguirre‐Gutiérrez et al. 2025; Martínez‐Villa et al. 2024), which could ultimately affect their current strong carbon sequestration. However, it remains unclear whether montane and lowland tree species differ in their physiological thermal plasticity and whether the observed warming‐induced changes in species composition in the tropical Andes are driven by differences in thermal plasticity between these two species groups.

Photosynthesis and leaf dark respiration (*R*_d_) are vital physiological processes that strongly dictate the availability of organic carbon necessary for plant energy and growth (O'Leary et al. 2019; Reich et al. 2022). Therefore, these two physiological processes may potentially underlie the strong divergent growth responses to warming observed in montane versus lowland Andean tree species, although no study to date has directly explored this. The response of leaf dark respiration (*R*_d_) to long‐term warming among tropical trees is relatively well characterized; recent studies on both tropical montane and lowland tree species have consistently shown that leaf dark respiration strongly acclimates to warming, resulting in broadly similar respiration rates at prevailing growth temperatures between control and warmed plants (Cheesman and Winter 2013a, 2013b; Choury et al. 2022; Drake et al. 2015; Dusenge et al. 2021; Mujawamariya et al. 2021; Scafaro et al. 2017; Slot and Winter 2017, 2018; Zhu et al. 2021). A recent experimental field study on tropical Andean forest tree species also showed similar thermal acclimation response patterns of leaf *R*_d_ between montane and lowland (Cox et al. 2023), indicating that leaf respiration is not expected to impose a substantial limitation on carbon available for tree growth as the climate warms. However, a detailed assessment of photosynthetic thermal plasticity in tropical montane and lowland tree species under realistic field conditions is lacking (Feeley et al. 2023; Wu et al. 2025).

**FIGURE 1:**
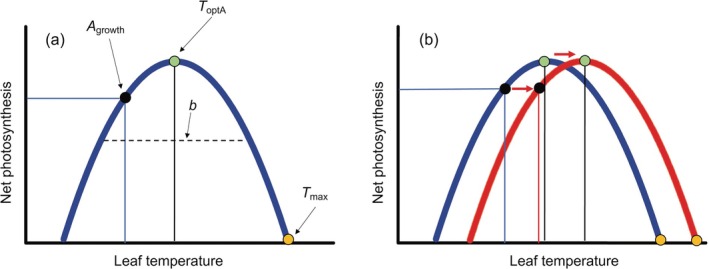
Conceptual figure of a constructive adjustment of net photosynthesis in response to increasing growth temperature. (a) Key parameters that are used to examine the thermal adjustment of net photosynthesis are shown, including net photosynthesis at growth temperature (*A*_growth_), the thermal optimum of net photosynthesis (*T*_optA_), the b parameter, which represents the breadth of the temperature response of net photosynthesis, and the high‐temperature CO_2_ compensation point (*T*_max_). (b) Hypothesized responses of a constructive adjustment of net photosynthesis in response to warming in which, simultaneously, the *T*_optA_ and *T*_max_ shift to higher leaf temperatures and *A*_growth_ is either maintained or increased with an increase in growth temperature. This figure assumes that the breadth of the curve is not altered by growth temperature. Redrawn from Way and Yamori (2014).

Photosynthetic thermal acclimation is commonly characterized by changes in parameters related to the thermal sensitivity of rates of net photosynthesis and underlying processes, including apparent maximum rate of Rubisco carboxylation (*V*_cmax_) and the maximum rate of electron transport (*J*_max_) (Crous et al. 2022; Hikosaka et al. 2006; Kumarathunge et al. 2019; Way and Oren 2010; Yamori et al. 2014). The thermal sensitivity parameters of net photosynthesis include the thermal optimum of net photosynthesis (*T*_optA_), the high‐temperature CO_2_ compensation point (*T*_max_), and the breadth of the temperature response (*b*) (Figure 1a), whereas those of apparent *V*_cmax_ and *J*_max_ include their respective thermal optima, *T*_optVcmax_ and *T*_optJmax_, and activation energies, *E*_aVcmax_ and *E*_aJmax_ (Kumarathunge et al. 2019). The majority of tropical studies on photosynthetic thermal acclimation have focused on the effects of warming on the rates of *A*_net_, as well as on rates of apparent *V*_cmax_ and *J*_max_, with results varying across studies—with some showing increases, others showing decreases, or no change (Cheesman and Winter 2013b; Choury et al. 2022; Cox et al. 2023; Crous et al. 2023, 2025; Drake et al. 2015, 2024; Dusenge et al. 2021; Mujawamariya et al. 2023; Scafaro et al. 2017; Slot and Winter 2017, 2018; Smith and Dukes 2017; Wittemann et al. 2022). However, fewer tropical studies have focused on the thermal responses of photosynthetic sensitivity parameters, and all of these studies were mostly conducted on seedlings grown for less than 6 months (Choury et al. 2022; Dusenge et al. 2021; Slot and Winter 2017; Wittemann et al. 2022). The latter studies also reported high variability in the shift of *T*_optA_, with some species showing significant shifts (0.35°C–0.47°C per 1°C) (Choury et al. 2022; Slot and Winter 2017; Wittemann et al. 2022), whereas others showed a nonsignificant shift in *T*_optA_ (Crous et al. 2025; Dusenge et al. 2021; Wittemann et al. 2022). Only a handful of these studies focused on montane tree species and reported mixed responses, with the majority showing no significant shift in *T*_optA_ (Dusenge et al. 2021; Wittemann et al. 2022). Clearly, more data are still needed on the thermal plasticity of photosynthesis in tropical tree species, particularly on trees growing under realistic field conditions and over longer experimental timescales.

Perfect thermal acclimation of photosynthesis should ideally involve two main responses to warming: a positive shift in the thermal optimum of net photosynthesis (*T*_optA_) and the concurrent maintenance or improvement of net photosynthetic rates at prevailing growth temperatures (*A*_growth_). This dual response is termed *constructive adjustment* (Way and Yamori 2014) (Figure 1). Consequently, constructive adjustment in photosynthesis, coupled with widespread thermal acclimation of leaf respiration (Cox et al. 2023; Crous et al. 2022; Mujawamariya et al. 2021; Reich et al. 2016; Slot and Kitajima 2015; Zhu et al. 2021), should potentially result in similar or improved plant growth rates across different thermal environments, assuming other key abiotic (e.g., nutrients, water, and light) (Fatichi et al. 2014; Körner 2015; Reich et al. 2018) and biotic factors (e.g., competition and symbiotic relationships) (Aerts 1999; Alexander et al. 2015; Zhou et al. 2022) are not limiting, and that gained carbon is mainly allocated to tree growth (Dusenge et al. 2021; Way and Oren 2010; Zhou et al. 2022). In a recent 5‐year field experiment at the boreal‐temperate ecotone, the thermal responses of net photosynthetic rates were found to correlate with tree growth and survival in response to warming across nine boreal and temperate tree species (Reich et al. 2022). However, no study to date has assessed whether potential thermal acclimation in photosynthesis, through shifts in *T*_optA_ and changes in *A*_growth_, is coupled with growth in tropical tree species, or whether these responses differ between montane and lowland species when both groups are exposed to changes in thermal conditions under realistic field conditions.

In this study, we assessed the thermal acclimation of net photosynthesis (*A*_net_) and its underlying processes, including apparent *V*_cmax_ and *J*_max_, in ~5‐year‐old juvenile trees of 13 dominant tropical Andean species. These trees were grown for 3 years across three common plantations along an elevation gradient in the northwestern range of Colombian Andes. We also examined the relationship between net photosynthesis rates at prevailing growth temperature (*A*_growth_) and relative growth rate. Nine species were of montane origin with a mean temperature of their geographic distribution around 14°C (hereafter referred to as “14°C‐montane”), whereas four species of lowland origin: two species with a mean temperature of their geographic distribution around 22°C (hereafter “22°C‐lowland”), and two with respective mean temperature of around 26°C (hereafter “26°C‐lowland”) (Figure S1). The three experimental plantations differed in mean annual air temperature (14°C, 22°C, and 26°C), closely aligned to the three mean temperatures of the species' distributions. Therefore, the “14°C‐montane” species, experienced warming when planted at the 22°C site, which also represents the hot extreme temperature. In contrast, the “22°C‐lowland” experienced warming when planted at the 26°C site but cooling at the 14°C site, mimicking their upslope migration during thermophilization. However, the 14°C site represent the coldest range of the lowland species. Similarly, the “26°C‐lowland” were cooled when planted at both the 22°C and 14°C sites (Cox et al. 2023; Restrepo et al. 2025). Although the “14°C‐montane” species were also planted at the 26°C site, no tree species survived at this site, likely due to significant deviation of growth temperatures from their natural distribution. Overall, our study offers some insight on physiological mechanisms that may potentially underly observed thermophilization process in the Andes.

For this study, we hypothesized that:

- montane Andean tree species are expected to exhibit weaker thermal acclimation of photosynthesis than lowland species, due to the widely observed thermophilization (Duque et al. 2015) and the previously documented weak thermal acclimation in African tropical montane seedlings (Dusenge et al. 2021; Wittemann et al. 2022);
- *A*_growth_ rates would correlate strongly with tree growth.

## 2 Materials and Methods

### 2.1 Site Description and Experimental Design

This study is part of an ongoing, long‐term project investigating the impact of warming on 15 of the most dominant species in Colombian tropical montane forests (Cox et al. 2024, 2023; Restrepo et al. 2025). We selected tree species from either montane or lowland provenances, which are naturally adapted to different temperature conditions. To classify species in these two provenances, we used natural tree distribution data from the Botanical Information and Ecology Network (Maitner et al. 2018) and associated temperatures from the high resolution (1 km^2^) global temperature dataset (Fick and Hijmans 2017; Restrepo et al. 2025). Nine species were montane with a mean temperature of their geographic distribution of 13.7°C, and four lowland species: two species with a mean temperature of 22.02°C, and two with a mean temperature of 25°C (Figure S1). Three mixed‐species plantations were established at three different sites along an elevational gradient. Due to the natural covariation of elevation and ambient temperature, the three sites also differ in the ambient temperature experienced by plants in the field (Table S1), offering an excellent opportunity to study the impact of warming under realistic field conditions. Therefore, the site names used in this paper refer to the mean annual temperature of each site: 14°C (high elevation), 22°C (moderate elevation), and 26°C (low elevation) (Table 1).

**TABLE 1:** Summary report of ANOVA showing degrees of freedom, *F*‐values, *p* values.

		df	df_res_	*F*	*p*
*T*_optA_	Site	2	20	17.1	**< 0.0001**
	Provenance	2	20	1.8	0.19
	Site: Provenance	3	20	3.2	**0.042**
*b*	Site	2	20	7.9	**0.0028**
	Provenance	2	20	1.1	0.4
	Site: Provenance	3	20	0.5	0.7
*T*_max_	Site	2	20	3.7	**0.044**
	Provenance	2	20	0.06	0.9
	Site: Provenance	3	20	0.44	0.7
*A*_growth_	Site	2	20	1.6	0.23
	Provenance	2	20	0.5	0.6
	Site: Provenance	3	20	0.9	0.4

*Note:* Thermal optimum of net photosynthesis (*T*_optA_, °C); *b* parameter (unitless); high‐CO_2_ compensation point (*T*_max_, °C); rates of net photosynthesis at growth temperature (*A*_g_, μmol m^−2^ s^−1^). Bold numbers represent *p*‐value less than 0.05 (*p* < 0.05).

Before planting trees in the experiment plantations, seeds from all species were collected from a montane rainforest near the 14°C experimental site, at elevations ranging from 1300 to 2500 masl. We chose to collect all seeds from a single location to minimize intraspecific variation that may be driven by adaptation to local environmental conditions at each of the three sites. The seeds of montane species were collected at elevations between 2200 and 2500 masl, where temperatures are very close to their optimal growth range, whereas seeds of lowland species were collected at elevations between 1300 and 2200 masl, where temperatures are at the coldest end of their natural distribution range. To further minimize intraspecific variation, seeds were collected from three to five trees per species. The seeds were subsequently propagated in polypots for 8–24 months (due to species‐specific differences in germination rate) in a nursery located near the 22°C site. At the time of planting from November to December 2018, the height of plants varied between 50 and 100 cm across different species. The saplings were transplanted in soils across the three experimental plantations with common soil (400 kg per tree) collected near the 14°C site. This soil had similar chemical characteristics to the soil found in the forest adjacent to the 14°C site. Each site consisted of four 600 m^2^ plots, and each plot was further subdivided into six 94 m^2^ blocks, with one individual from each species planted in each block (15 species × 6 blocks × 4 plots = 360 plants per site) (Aguirre‐Gutiérrez et al. 2025; Cox et al. 2024, 2023). One of the blocks was fertilized every 4 months to provide similar soil nutrient content, planted 2.5 m apart from each other to avoid competition. However, in this study we measured trees of 13 species (nine montane and four lowland species; Figure S1) that were grown in unfertilized blocks. The unfertilized soil used was fertile soil without soil nutrient limitations to the growth of plants. As precipitation differs across sites (Table S1), trees were regularly watered (an average of 8–24 L of water per tree per night were used after no rain events during two consecutive days) to avoid water limitations. Specific details of fertilizer addition, including quantities and timings, the nutrient composition of soils at the native forest where seeds were collected, the original soils at experimental sites, the soils used for planting, and the maximum number of consecutive days without rain at experimental sites are provided in Restrepo et al. (2025).

### 2.2 Gas Exchange Measurements

Leaf gas exchange measurements were conducted between January and March 2022, approximately 3 years after transplanting saplings into the experimental plantations. One healthy (i.e., green without visible damage from herbivores) leaf from each of four saplings of each species (i.e., one individual per plot) at each site was measured with one portable photosynthesis system (Li‐Cor 6800; Li‐Cor Inc., Lincoln, NE, USA). Light‐saturated net CO_2_ assimilation rates (*A*_n_) were measured at varying intercellular CO_2_ concentrations (*C*_*i*_), producing so‐called *A* − *C*_*i*_ curves. These *A* − *C*_*i*_ curves were conducted at a PPFD (photosynthetic photon flux density) of 1800 μmol photons m^−2^ s^−1^, the airflow rate of 750 μmol s,^−1^ and at five leaf temperatures (15°C, 20°C, 25°C, 30°C, 35°C and 40°C) using the 6 cm^2^ 6800‐01A Fluorometer chamber. The leaf cuvette temperature was controlled using the instrument's leaf thermocouple. However, due to the Li‐Cor 6800's leaf temperature control, which operates within 10°C around the ambient temperature, we were able to conduct measurements ranging from 15°C to 35°C at both 14°C and 22°C, whereas measurements at the 26°C site were limited to a 20°C*–*40°C temperature range. The *A* − *C*_*i*_ curve was launched when gas exchange was stable at the reference CO_2_ concentration of 410 μmol m^−2^; CO_2_ concentrations were then changed sequentially to 410, 50, 100, 150, 250, 410, 800, 1200, 1600, and 2000 μmol mol^−1^. Before starting any *A* − *C*_*i*_ curve, the leaf was allowed to acclimate at each temperature for at least 10–15 min, and the *A* − *C*_*i*_ was initiated once both net photosynthesis and stomatal conductance were stable for at least 2 min. Throughout each *A* − *C*_*i*_, the stability time at each CO_2_ reference concentration was set to 45–180 s, and the automatic match was programmed before recording any data at each CO_2_ reference concentration. The Li‐Cor 6800 indicates whether there is a leak, and measurements were always done after ensuring there were no leaks in the system. Therefore, no subsequent leak correction was necessary. At the beginning of each measurement day, an automatic warm‐up test was run to detect any problems within the instrument, and only measurements were initiated when all errors had been fixed, as suggested by the instrument operation protocol. After the data quality check, a total of 92 temperature responses of *A* − *C*_*i*_ curves were used in this study (Dusenge et al. 2024).

### 2.3 Parameterisation

The C_3_ photosynthesis model—FvCB (Farquhar, von Caemmerer, and Berry) (Farquhar et al. 1980) was used to parameterize *V*_cmax_ and *J*_max_ from the *A* − *C*_*i*_ curves using the *fitacis* function from the plantecophys 1.4‐6 R package (Duursma 2015) using the *bilinear* fitting method. The temperature dependencies of the CO_2_ compensation point in the absence of mitochondrial respiration (Γ*) and the Michaelis—Menten constants for CO_2_ and O_2_ (*K*_c_ and *K*_o_) were taken from (Bernacchi et al. 2001). The leaf mesophyll conductance for CO_2_ was not measured, so the parameterized *V*_cmax_ and *J*_max_ are considered “apparent” rather than “true” values, because they are based on intercellular CO_2_ concentrations (*C*_*i*_), rather than the CO_2_ concentration at the site of carboxylation (*C*_c_) in the chloroplast. We also did not attempt to assume any value for *g*_m_ due to its dependence on internal leaf anatomical traits, as well as daytime foliar respiration—all of which may potentially differ among the studied 13 species and are inherently difficult to measure, particularly in remote field conditions such as these (Cousins et al. 2020; Evans 2021; Tcherkez et al. 2017). Moreover, *g*_m_ responses to temperature vary widely among the few studied C_3_ species, and no widely accepted formulation currently captures this temperature sensitivity (Evans 2021). Therefore, our analyses are based on “apparent” values of *V*_cmax_ and *J*_max_, rather than the “true” values at the carboxylation site within the chloroplasts, as is commonly done in other empirical studies, including those conducted at remote field sites (Crous et al. 2025; Mujawamariya et al. 2023; Sibret et al. 2025; Vårhammar et al. 2015) and used in most large‐scale modelling (Friedlingstein et al. 2025; Mercado et al. 2018; Oliver et al. 2022; Sitch et al. 2024) studies. Subsequently, we parameterized the temperature sensitivity parameters of apparent *V*_cmax_ (*T*_optV_ and *E*_aV_) and *J*_max_ (*T*_optJ_ and *E*_aJ_) using the modified Arrhenius function outlined in the following Equation (1) (Medlyn et al. 2002):

(1) $$f\left(T_{k}\right)=k_{\mathrm{opt}}\frac{H_{d}\exp\left(\frac{E_{a}\left(T_{k}-T_{\mathrm{opt}}\right)}{T_{k}RT_{\mathrm{opt}}}\right)}{H_{d}-E_{a}\left(1-\exp\left(\frac{H_{d}\left(T_{k}-T_{\mathrm{opt}}\right)}{T_{k}RT_{\mathrm{opt}}}\right)\right)}$$

*k*_opt_ is the rate of apparent *V*_cmax_ or *J*_max_ at the optimum temperature (*V*_cmaxopt_, *J*_maxopt_), *H*_d_ (kJ mol^−1^) is the deactivation energy term that describes the decline in enzyme activity at higher temperature, *E*_a_ (kJ mol^−1^) is the activation energy term that describes the exponential increase in enzyme activity with an increase in temperature, *R* is the universal gas constant (8.314 J mol^−1^ K^−1^), and *T*_opt_ and *T*_k_ are the optimum and given temperatures of the apparent *V*_cmax_ or *J*_max_. To avoid over‐parameterisation, the value of *H*_d_ was fixed at 200 kJ mol^−1^ (Medlyn et al. 2002). We also used Equation (1) to calculate *V*_cmax_ and *J*_max_ at the mean daytime growth temperature of each site: 21.4°C, 24.3°C, and 31.2°C, for the 14°C, 22°C, and 26°C sites, respectively (Table S2). These growth temperatures were calculated as mean daytime (6 a.m.–6 p.m.) temperatures for 1 month before each site's measurement campaign.

Net photosynthesis data at 410 ppm were extracted from each *A* − *C*_*i*_ curve and used to fit Equation (2) (Gunderson et al. 2010) to parameterize the thermal optimum of net photosynthesis (*T*_optA_).

(2) $$A\left(T\right)=A_{\mathrm{opt}}-b{\left(T-T_{\text{optA}}\right)}^{2}$$

where *A* (*T*) is the *A* (μmol m^−2^ s^−1^) at a given air temperature *T* (°C), *A*_opt_ is the *A* at the optimum temperature (*T*_opt_), and the *b* parameter represents the breadth of the photosynthetic temperature response curve; larger values of *b*, that is, lower breadth, indicate that *A* (*T*) has greater sensitivity to changes in *T*. After fitting *b* and *T*_opt_, we used Equation (2) to model net photosynthesis at prevailing daytime growth temperatures (21.4°C, 24.3°C, and 31.2°C for 14°C, 22°C, and 26°C site, respectively) conditions (*A*_growth_) (Table S2).

The high‐temperature CO_2_ compensation point (*T*_max_) was estimated by rearranging Equation (2) as follows:

(3) $$T_{\max}={\left(\frac{A_{\mathrm{opt}}}{b}\right)}^{0.5}+T_{\text{optA}}$$

We also estimated whether stomatal conductance and leaf respiration may have affected the shifts in *T*_optA_ in response to warming. To explore the role of stomatal conductance, we re‐calculated net photosynthesis at a *C*_*i*_/*C*_a_ ratio of 0.7 (with a final *C*_*i*_ of 287 μmol mol^−1^) which removes any potential CO_2_ diffusional limitations, and we derived the thermal optimum of net photosynthesis but at a common *C*_*i*_ of 287 μmol mol^−1^ using Equation (2) (*T*_opt287_). To examine the potential role of respiration, we re‐calculated gross photosynthesis by not subtracting the *R*_day_ (respiration during the light) term in Equations (4) and (5) (von Caemmerer 2000) below, and we then estimated the thermal optimum of gross photosynthesis using Equation (2).

(4) $$A_{c}=\frac{V_{\text{cmax}}\left(C_{i}-\Gamma^{*}\right)}{\left[C_{i}+K_{c}\left(1+\frac{O}{K_{O}}\right)\right]}-R_{\mathrm{day}}$$

where *O* is the intercellular concentration of O_2_, *K*_c_ and *K*_o_ are the Michaelis–Menten coefficients of Rubisco activity for CO_2_ and O_2_, respectively, and Γ* is the CO_2_ compensation point in the absence of mitochondrial respiration. Values at 25°C and temperature sensitivities of Γ*, *K*_c_, and *K*_o_ were taken from Bernacchi et al. (2001).

(5) $$A_{j}=\left(\frac{J_{\max}}{4}\right)\times \frac{\left(C_{i}-\Gamma^{*}\right)}{\left(C_{i}+2\Gamma^{*}\right)}-R_{\mathrm{day}}$$

*A* was considered as the minimum of *A*_c_, and *A*_j_.

Stomatal conductance at growth temperature (*g*_sTg_) was retrieved from the *A* − *C*_*i*_ curves at leaf temperatures of 20°C, 25°C, and 30°C for the 14°C, 22°C, and 26°C sites, respectively. These leaf temperatures were approximately close to mean daytime prevailing growth temperatures of 21.4°C, 24.3°C, and 31.2°C, respectively, for the month prior to our field campaign (Table S2).

### 2.4 Relative Growth Rates

Quarterly measurements on tree diameter were taken from February 2019 until January 2022 (Restrepo et al. 2025; Restrepo and Mercado 2024). The tree diameter (*D*) was used to calculate the relative growth rate (RGR—a metric that indicates the proportion changes of growth per unit of time) as the difference between the logarithm of the tree diameter at *i* census (*D*_*i*_) and the diameter taken during the first census (*D*_0_) divided by the time interval between measurements (*t*_*i*_ – *t*_0_): The growth rate per tree is derived as follows (Restrepo et al. 2025):

(6) $$\mathrm{RGR}=\left[\log\left(D_{i}\right)-\log\left(D_{0}\right)\right]/\left(t_{i}-t_{0}\right)$$

With *D* expressed in millimeters (mm), (*t*_*i*_ – *t*_0_) in years, and RGR in mm mm^−1^ year^−1^.

### 2.5 Statistical Analyses

To analyze the effect of growth temperature on all the measured traits, we used two‐way ANOVA with site and thermal affiliation (montane, lowland 22°C, and lowland 22°C) as the main factors and used the mean value of each species and site. The significance difference was assessed at a *p* value threshold of 0.05. We tested whether homogeneity of variance and normality assumptions were met using the Levene and Shapiro–Wilk tests, respectively. We further used post hoc Dunn‐Sidak's significance tests to evaluate differences among sites and affiliation by using *emmeans* (Lenth 2024) and *multcomp* (Hothorn et al. 2008) R packages. The correlation analyses between traits were done using simple linear regression. At last, to test the effects of stomatal conductance and leaf respiration, we used unpaired *t*‐tests to compare observed *T*_opt_ and thermal optima values (i) obtained after removing the effect of stomatal conductance (i.e., at a common *C*_*i*_) and (ii) *T*_opt_ of gross photosynthesis (which include leaf respiration). All analyses were performed in R version 4.3.1.

## 3 Results

### 3.1 Thermal Acclimation of Photosynthesis

**FIGURE 2:**
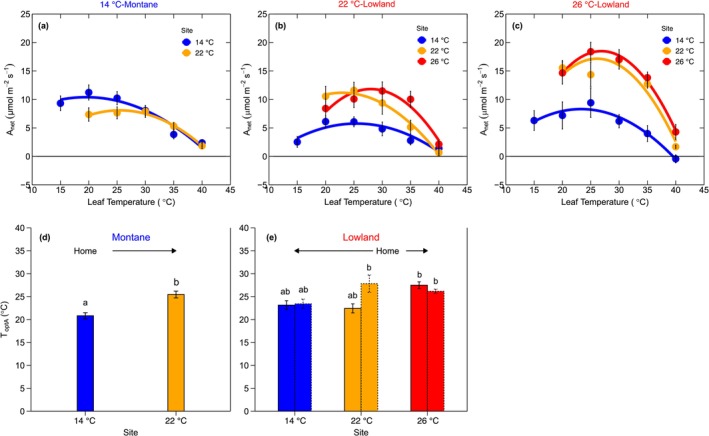
Temperature responses of net photosynthesis. Panels (a–c) represent the temperature response of net photosynthesis for montane (a), lowland native at the 22°C site (b), and lowland native at the 26°C site (c). Colors represent mean annual temperature at experiment sites (14°C = blue; 22°C = orange; 26°C = red). Each data point represents the mean value (mean ± SE) of all biologically independent trees across all species at each leaf temperature, group, and site (Montane: *N* = 15–18 trees; Lowland 22°C: *N* = 5–8 trees; Lowland 26°C: *N* = 4–6 trees). The data were fitted with the Equation (2). Panels (d, e) represent the optimum temperature of net photosynthesis (*T*_optA_, °C) at different sites for montane (d) and lowland (e) tree species, respectively. The *x* axis represents each site's mean annual temperature: 14°C, 22°C, and 26°C (Table S1). ‘Home’ indicates the native thermal environment for each species group (14°C for montane; 22°C–26°C for lowland), and arrows indicate whether species were subjected to warming or cooling. The solid and dashed bars in (e) represent lowland species originating from the 22°C and 26°C sites, respectively. Small letters are used for statistical comparisons among each species group and site combination, where a different letter denotes a significant difference at the *p* < 0.05 threshold from *Sidak* posthoc test. Further details on statistical analyses for this figure can be found in Table 1.

The optimum temperature of net photosynthesis (*T*_optA_) adjusted to changes in growth temperature (Figure 2; Table 1; Table S3). In 14°C‐montane tree species growing at 22°C, *T*_optA_ significantly shifted by 0.6°C per 1°C warming (Figure 2a,d). The 22°C‐lowland species growing at the warmest site (26°C) shifted their *T*_optA_ by 1.2°C per 1°C of warming (Figure 2b,e). By contrast, in all lowland species growing at cooler sites (i.e., 22°C‐lowland species growing at 14°C, and 26°C‐lowland species growing at both 14°C and 22°C), *T*_optA_ either remained unchanged or decreased weakly (Figure 2b,c,e and Table 1). Specifically, cooling induced no significant shift in the 22°C‐lowland species when grown at the 14°C site, whereas the 26°C‐lowland species exhibited no shift when grown at the 22°C, and there was a small but nonsignificant shift of ~0.23°C per 1°C cooling when grown at 14°C (Figure 2e). Surprisingly, at the intermediate growth temperature of 22°C, montane and lowland species had similar *T*_optA_, around 25.3°C, indicating that montane species strongly acclimated to this warmer growth condition to achieve similar photosynthetic performance as the lowland species in this novel thermal environment.

In montane species, the thermal optima of apparent *V*_cmax_ and *J*_max_, *T*_optV_ and *T*_optJ_, respectively, also strongly adjusted to warming (Figure S2). Specifically, both *T*_optV_ and *T*_optJ_ significantly increased by 0.7°C per 1°C warming (Figure S2; Tables S4 and S5). In the 22°C‐lowland species grown at the 26°C site, there was no significant shift in *T*_*o*ptV_ and *T*_optJ_. Similarly, the shifts in *T*_optV_ and *T*_optJ_ were also nonsignificant for lowland species under cooling (Figure S2; Table S4). As a result, *T*_optA_ was positively correlated with both *T*_optV_ and *T*_optJ_, whereas in lowland species, such relationships were absent (Figure S3). However, in both groups, shifts in *T*_optA_ were not linked to changes in the ratio of *J*_max_ to *V*_cmax_ (Figure S4), the activation energy of *V*_cmax_ (*E*_aV_) and *J*_max_ (*E*_aJ_) (Figure S5), CO_2_ diffusion through stomata or leaf respiration (Table S6).

**FIGURE 3:**
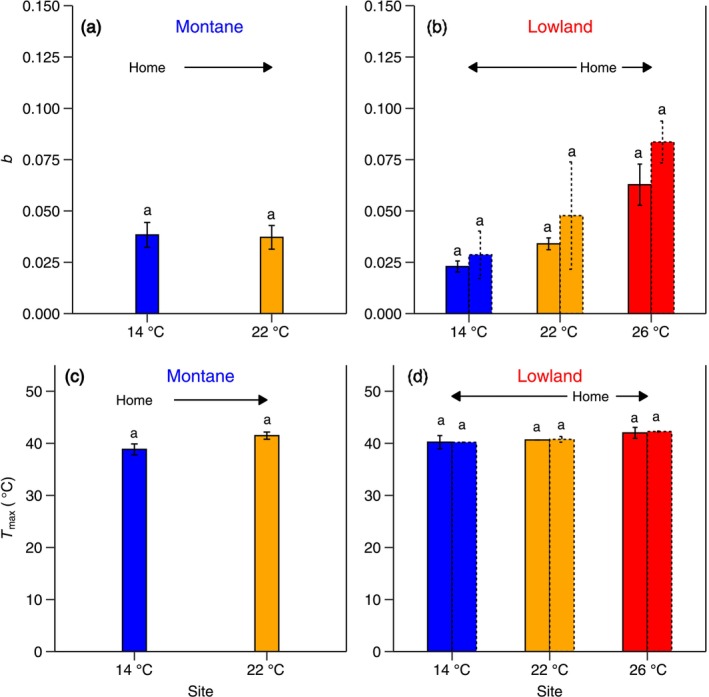
Key temperature sensitivity parameters of net photosynthesis. The impact of growth temperature on the *b* parameter that represents the breadth of the temperature response curve of net photosynthesis measured at ambient CO_2_ concentration in montane (a) and lowland (b) tree species. The impact of growth temperature on the upper leaf temperature at which net photosynthesis rates measured at ambient CO_2_ are zero (*T*_max_, °C) in montane (c) and lowland (d) tree species. The *x* axis represents each site's mean annual temperature: 14°C, 22°C, and 26°C (Table S1). ‘Home’ indicates the native thermal environment for each species group (14°C for montane; 22°C–26°C for lowland), and arrows indicate if they were subjected to warming or cooling. The solid and dashed bars in (b, d) represent lowland species originating from the 22°C and 26°C sites, respectively. Small letters are used for statistical comparisons among each species group and site combination, where a different letter denotes a significant difference at the *p* < 0.05 threshold from *Sidak* posthoc test. Further details on statistical analyses for this figure can be found in Table 1.

The spread of the temperature response of net photosynthesis represented by parameter *b* (Figure 1a) was affected by growth temperature (Table 1; Table S3). Although no statistically significant differences were observed between the two groups, two distinct patterns emerged in their responses of the *b* parameter (Figure 2a–c). In montane species, *b* was overall lower (i.e., broad curves, and thus lower thermal sensitivity; Figure 2a) and remained unchanged across sites (Figure 3a). However, in the 22°C‐lowland species, parameter *b* increased by 85% in trees growing at the 26°C site (Figure 2b,c; Figure 3b; Table 1; Table S3). With respect to cooling, *b* in the 22°C‐lowland trees grown at the 14°C site decreased by 33% (Figure 3b), whereas *b* in the 26°C‐lowland species grown at the 22°C and 14°C sites decreased by 43% and 66%, respectively (Figure 3b). Altogether, these results suggest that montane species exhibited consistently broad temperature response curves for net photosynthesis irrespective of the growth thermal environment, whereas lowland species showed broad curves only when growing in higher elevation, cooler sites compared to their warmer, native habitats in lower elevations (Figure 2a–c).

The upper leaf temperature threshold at which net photosynthesis rates become zero or the so‐called high temperature CO_2_ compensation point (*T*_max_) (Figure 1a) exhibited an increasing trend from high‐ to low‐elevation sites in both groups, but none of these shifts were significant in either group (Figure 3c,d; Table 1; Table S3).

### 3.2 Photosynthesis at Growth Temperatures

**FIGURE 4:**
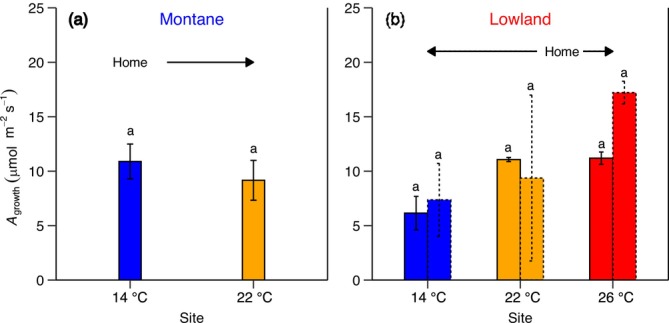
Net photosynthetic rates at growth temperature. The impact of growth temperature on the rates of net photosynthesis measured at ambient CO_2_ and prevailing growth temperature (*A*_growth_, μmol m^−2^ s^−1^) in (a) montane and (b) lowland tree species. The *x* axis represents each site's mean annual temperature: 14°C, 22°C, and 26°C (Table S1). However, estimation of *A*_growth_ rates was done using mean daytime (6 a.m.–6 p.m.) growth temperature (14°C site = 21.4°C, 22°C site = 24.3°C, and 26°C = 31.2°C) of 1 month prior to each measurement campaign at each site, the time defined as the thermal acclimation period of photosynthesis. “Home” indicates the native thermal environment for each species group (14°C for montane; 22°C–26°C for lowland), and arrows indicate if they were subjected to warming or cooling. The solid and dashed bars in (b) represent lowland species originating from the 22°C and 26°C sites, respectively. Small letters are used for statistical comparisons among each species group and site combination, where a different letter denotes a significant difference at the *p* < 0.05 threshold from *Sidak* posthoc test. Further details on statistical analyses for this figure can be found in Table 1.

Net photosynthetic rates at prevailing growth temperature (*A*_growth_) were not significantly different between montane and lowland species, and across different sites largely masked by high interspecific variations (Figure 4; Table 1; Table S7). Specifically, montane species largely maintained constant rates of *A*_growth_ across 14°C–22°C sites, despite an ~8°C increase in growth temperature. Similarly, the 22°C‐lowland species also achieved homeostasis in *A*_growth_ rates between the 22°C and 26°C sites (Figure 4b). In contrast to warming, cooling resulted in a 44% decrease in *A*_growth_ rates in the 22°C lowland species when grown at the 14°C site. Cooling also reduced *A*_growth_ rates in 26°C‐lowland species by 46% and 57% at the 22°C and 14°C sites, respectively (Figure 4b). In montane species, constant rates in *A*_growth_ were correlated with both similar constant rates in CO_2_ supply through stomata (Figure S6a) and apparent photosynthetic capacity (Figure S7a,c; Figure S3). Similarly, in lowland species, changes in *A*_growth_ strongly mirrored those of apparent photosynthetic capacity across the sites, especially for apparent *V*_cmax_ (Figure S7b,d; Table S7). Overall, these findings suggest that with constant *A*_growth_ rates, montane species successfully maintain homeostasis in carbon uptake across different thermal environments. In contrast, the decrease in *A*_growth_ for lowland species with cooling indicates strong limitation by apparent Rubisco carboxylation at higher elevations, which leads to an overall reduction in physiological performance as they move to higher elevations and cooler environments.

As photosynthesis is strongly related to leaf nitrogen on area basis (*N*_a_) (Ellsworth et al. 2022) with the latter usually affected by growth temperature (Crous et al. 2022), leaf *N*_a_ is expected to mediate species responses of net photosynthesis to warming (Dusenge et al. 2020, 2021). In our study, although leaf nitrogen (*N*_a_) did not significantly differ overall among sites, it showed a decreasing trend in warmer (lower elevations) environments in both groups (Figure S8; Table S8). However, leaf *N*_a_ was significantly higher in lowland species compared to montane counterparts (Figure S8; Table S4). This difference in leaf *N*_a_ between species groups was expected as all lowland species used in this study are from the *Inga* genus, which is well known to form symbiotic associations with nitrogen‐fixing bacteria (Eaton and Hamilton 2023). Therefore, we standardized *A*_growth_ to leaf nitrogen (*A*_gN_) to investigate whether changes in *A*_growth_ responses were mediated by leaf *N*_a_. In both groups, the warming response patterns of *A*_gN_ were similar to the nonstandardized values (*A*_growth_) (Figure S9; Table S3), suggesting that leaf *N*_a_ did not play a significant role in the thermal responses of net CO_2_ uptake in our studied species.

### 3.3 Photosynthesis and Tree Growth Rate

**FIGURE 5:**
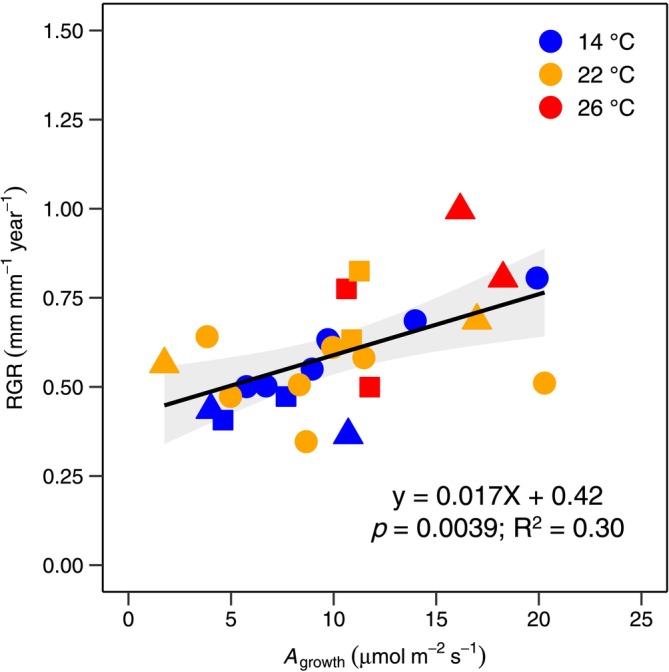
Relationship of net photosynthesis and relative growth rates. The relative growth rate (RGR, mm mm^−1^ year^−1^) as a function of rates of net photosynthesis measured at ambient CO_2_ and growth temperature (*A*_growth_, μmol m^−2^ s^−1^). Colors represent mean annual temperature of experiment sites (14°C = blue; 22°C = orange; 26°C = red). A simple linear regression model was used to analyse the relationship between these two variables across species and groups. Each data point represents each species mean. The statistical test was one‐sided since it was done to evaluate whether there is a positive relationship between the two variables. The solid black line represents the regression line, and the light gray area represent 95% confidence interval around the regression line.

*A*_growth_ and relative tree growth rates were strongly correlated (Figure 5). Our results showed that *A*_growth_ explained about 30% of the relative growth rate across montane and lowland tree species. This positive relationship suggests that growth in these Andean tree species is largely influenced by changes in net carbon uptake.

## 4 Discussion

Some tropical plant communities are currently experiencing negative impacts from ongoing climate warming (Cuni‐Sanchez et al. 2024; Duque et al. 2015; Hubau et al. 2020; Ntirugulirwa et al. 2023), with tropical montane ecosystems generally facing the fastest rates of warming (Mata‐Guel et al. 2023) and associated tree mortality (Duque et al. 2015; Fadrique et al. 2018; Ntirugulirwa et al. 2023). However, there is still limited understanding of the physiological thermal plasticity of tropical tree species when growing under realistic field conditions (Feeley et al. 2023; Paulick et al. 2017). To reduce this knowledge gap, we examined the thermal plasticity of net photosynthesis, including its underlying photosynthetic capacity and its correlation with the relative growth rate in 5‐year‐old tropical montane and lowland saplings after a 3‐year exposure to sustained warming and cooling under field conditions at three sites along a natural temperature gradient at various elevations. For the first time, we demonstrate that Andean tropical montane tree species can acclimate their photosynthesis to warming to maintain a thermal optimum temperature for net photosynthesis (Figure 2) and net CO_2_ assimilation rates (Figure 4) similar to those of naturally co‐occurring lowland tree species. Across both montane and lowland tree species and thermal environments, rates of net photosynthesis at prevailing growth temperatures explained 30% of the relative growth rate after 3 years (Figure 5), indicating that thermal plasticity of photosynthesis is critical to tropical tree growth as the climate warms.

### 4.1 Thermal Plasticity of Photosynthesis

We demonstrate, for the first time, that tropical montane Andean tree species can acclimate their photosynthesis by significantly shifting their thermal optimum of net photosynthesis (*T*_optA_) by 0.6°C per 1°C warming to achieve a comparable *T*_optA_ with co‐occurring lowland species (Figure 2). This finding contradicts our first hypothesis, which was based on a handful of previous warming experiments conducted on potted seedlings of African tropical montane tree species. These experiments reported weak to no shifts in *T*_optA_ with warming (Dusenge et al. 2021; Wittemann et al. 2022). In contrast, our study, which is the first to explore thermal acclimation of photosynthesis on freely rooted juvenile trees of tropical montane tree species in realistic field conditions, indicates that tropical montane species can strongly shift their photosynthetic thermal optima to maintain constant rates of carbon assimilation in a warmer environment. The shift in *T*_optA_ for montane species is also notably greater than shifts reported in previous lowland studies, mostly done on potted seedlings (~0.47°C per 1°C) (Choury et al. 2022; Slot and Winter 2017). The shift in the thermal optimum of net photosynthesis in montane species was strongly correlated with the simultaneous thermal shifts in the thermal optima of apparent photosynthetic capacity (*V*_cmax_ and *J*_max_) (Choury et al. 2022; Dusenge et al. 2020; Wittemann et al. 2022), which also shifted by 0.7°C per 1°C warming (Figures S2 and S3). These findings indicate that the plasticity in the photosynthetic capacity is the strongest mechanism enabling these species to achieve such substantial shifts in *T*_optA_. Therefore, our study provides empirical evidence that montane Andean tree species possess the physiological capacity to adjust to the warming occurring in the Andes region. Some of the studied montane genera, such as *Ilex* and *Quercus*, have wide distribution ranges extending into the temperate region, which may partly explain the observed large thermal plasticity.

We also found that net photosynthetic temperature response curves in montane tree species remained broad across all sites. In contrast, curves in lowland species were broad only at the high‐elevation site (Figures 2 and 3). Our findings align with a long‐standing hypothesis, but which has never been validated by empirical data until now—that high‐altitude montane species should be adapted physiologically to larger diurnal temperature fluctuations than lowland tropical species (Ghalambor 2006). In high‐altitude tropical regions, daily temperature fluctuations are influenced by high daytime temperatures due to lower atmospheric pressure, the sun's vertical angle, low daytime temperatures resulting from frequent cloud cover, and typically low nighttime temperatures reflecting the higher elevation (Van De Weg et al. 2014). This contrasts with lowland areas, which generally experience warmer temperatures with less variation (Janzen 1967). Therefore, our study provides some evidence of these contrasting adaptations to diurnal temperature fluctuations between montane and lowland species. These results indicate that montane species are adapted to fix carbon over a wider range of temperatures, which vary significantly throughout the day in their native habitat. In contrast, lowland species fix carbon within a narrower, higher temperature range in their native habitat. However, when lowland species are grown at higher altitudes where temperatures fluctuate, they exhibit similarly broad curves as their montane counterparts. This indicates that lowland species may become less thermally sensitive when growing upslope under a warming climate.

### 4.2 Photosynthesis, a Driver of Tree Growth

We also observed that the strong thermal acclimation of the photosynthetic process enabled montane species to maintain homeostasis in net CO_2_ uptake under prevailing growth conditions, despite growing at the hot extreme of their range. This also enabled them to achieve net CO_2_ assimilation rates comparable to those of co‐occurring lowland species (Figure 4). Furthermore, we found a positive correlation between rates of net photosynthesis and relative growth (Figure 5), which supports our second hypothesis. Across all species, net photosynthesis explained 30% (*p* = 0.0039) of the relative growth rate after 3 years, indicating that growth in these tree species is significantly influenced by photosynthetic adjustment as temperature changes. Moreover, a recent study conducted after 6 months in the same experiment showed that leaf respiration strongly acclimated to warming, maintaining near homeostasis in rates of leaf respiratory CO_2_ losses with an increase in growth temperature (Cox et al. 2023). As leaf respiration has been shown to strongly acclimate to warming in tropical tree species (Cheesman and Winter 2013a, 2013b; Choury et al. 2022; Drake et al. 2015; Dusenge et al. 2021; Mujawamariya et al. 2021; Scafaro et al. 2017; Slot and Winter 2017, 2018; Zhu et al. 2021), our findings suggest that responses of leaf‐level net carbon gain to warming are a strong driver of tree growth in these Andean tree species. Similarly, a recent 5‐year warming field study on boreal and temperate species found that sapling growth and survival were strongly associated with photosynthetic responses to prevailing environmental conditions, including temperature and soil moisture (Reich et al. 2022). These findings emphasize the critical role of leaf physiological processes in the growth and survival of tree species as the climate warms. Many studies, including ours, focus on the response of sun‐exposed leaves. However, to fully understand the role of leaf physiological processes in influencing tree growth responses, we also need to determine whether shaded leaves, both inside and at the bottom of the canopy, thermally acclimate in the same way as sun‐exposed leaves. This knowledge will help us model how whole‐canopy net carbon gain contributes to whole‐tree growth rates in response to warming, potentially increasing the estimates reported in this study.

### 4.3 Broader Implications of Our Findings

Our current findings offer perspective on the ongoing thermophilization process in the Andes, which refers to the gradual changes in species composition, characterized by a relative increase in the abundance of lowland species at historically cooler, higher‐elevation sites. To date, no exact mechanisms are known to drive this thermophilization process. However, several main, non‐mutually exclusive processes could cause thermophilization with climate warming. The first process involves weak physiological plasticity in response to warmer growth conditions (Feeley et al. 2023). This could be primarily driven by heat and increased vapor pressure deficit associated with warming, potentially reducing carbon assimilation for plant growth and survival (Mirabel et al. 2023; Reich et al. 2022). Recent studies in tropical regions have suggested that weak physiological acclimation of photosynthesis to warming may contribute to observed thermophilization, although direct empirical evidence was lacking (Feeley, Martinez‐Villa, et al. 2020; Ntirugulirwa et al. 2023). Our study is among the first to examine the thermal plasticity of photosynthesis in naturally co‐occurring, dominant Andean montane and lowland tree species, aiming to shed light on whether photosynthesis may partially explain thermophilization. The results demonstrate that montane species can effectively acclimate their leaf‐level photosynthesis to maintain constant carbon assimilation rates across different thermal environments, rates that are also comparable to those of lowland counterparts (Figures 2a–c and 4). Based on these findings, we suggest that limitations in thermal acclimation of leaf‐level photosynthetic physiology are less likely to be among the main physiological drivers of thermophilization observed in the tropical Andes. However, considering the observed link between net photosynthesis and growth in our study, it is likely that the response of photosynthesis to other environmental drivers that change with climate warming (e.g., VPD, drought) strongly influences the observed thermophilization in the Andes. At last, although these leaf‐level responses provide fundamental insights into thermal responses of montane tree species, the responses of whole‐canopy leaf carbon assimilation (Dusenge et al. 2021) and plant biomass allocation strategies to various organs (leaves, branches, stems, and roots) under rising growth temperatures (Way and Oren 2010) may ultimately determine the competitiveness of montane tree species in a warming climate.

Other likely key drivers of thermophilisation are changes in species' competition for water, light, and nutrients, and dispersal modes of different species (Alexander et al. 2015; Duque et al. 2015). In our experiment, intraspecies competition was reduced early as seedlings were spaced 2.5 m from each other; however, species grow much closer in natural habitats, increasing competition for light and soil nutrients. Furthermore, warming is also associated with a decrease in cloud cover in the mountain regions (Hughes et al. 2024), which means that montane species in the future will be exposed to more frequent intense radiation than today, exposing trees to critical leaf temperature thresholds that affect optimal physiological functioning (Doughty et al. 2023). This scenario likely explains why no montane species survived at the low‐elevation (26°C) experimental site, as they were exposed to both constant extreme heat and intense light due to reduced cloud cover, unlike in their native environment. Therefore, future research must investigate how other key abiotic changes associated with climate change, such as drought and cloud cover (Hughes et al. 2024), and biotic changes, such as mycorrhizal associations (Zhou et al. 2022) and species competition (Alexander et al. 2015), might be involved in the thermophilisation process in the Andes. This understanding is crucial for informing sustainable conservation practices in these globally important Andean ecosystems. Therefore, we advocate for further research on other abiotic factors (e.g., thermal extremes, drought and changes in cloud cover) and biotic (e.g., competition and changes in symbiotic relationships) that may be driving thermophilisation. Nevertheless, our results suggest that the strong physiological acclimation of leaf‐level photosynthesis to warming alone among montane Andean tree species should be considered when predicting future impacts of warming on Andean plant communities.

## Author Contributions

**Mirindi Eric Dusenge:** data curation, formal analysis, investigation, methodology, visualization, writing – original draft, writing – review and editing. **Sebastian González‐Caro:** data curation, formal analysis, writing – review and editing. **Zorayda Restrepo:** conceptualization, data curation, methodology, project administration, writing – review and editing. **Anna Gardner:** data curation, formal analysis, writing – review and editing. **Patrick Meir:** conceptualization, funding acquisition, methodology, validation, writing – review and editing. **Iain P. Hartley:** conceptualization, funding acquisition, methodology, writing – review and editing. **Stephen Sitch:** conceptualization, funding acquisition, methodology, validation, writing – review and editing. **Adriana Sanchez:** data curation, funding acquisition, methodology, writing – review and editing. **Juan Camilo Villegas:** conceptualization, funding acquisition, methodology, writing – review and editing. **Lina M. Mercado:** conceptualization, funding acquisition, methodology, project administration, supervision, writing – review and editing.

## Conflicts of Interest

The authors declare no conflicts of interest.

## Supporting information


Data S1.

## Data Availability

The physiological data that support the findings of this study are openly available in Figshare at https://doi.org/10.6084/m9.figshare.29084276.v1. The tree growth data are available at NERC EDS Environmental Information Data Centre at https://doi.org/10.5285/c7ce1610‐aba3‐4a09‐bf7c‐1b6c774d597a.
