# Supplementary material for: Unexpected Large Photosynthetic Thermal Plasticity of Montane Andean Trees
Source: Glob Chang Biol. 2025 May 23;31(5):e70266. doi: 10.1111/gcb.70266 (PMC12100579; doi:10.1111/gcb.70266)
Supplement: Supplementary file 1 — Data S1. [file GCB-31-e70266-s001.pdf]

Unexpected large photosynthetic thermal plasticity of montane Andean trees

Mirindi Eric Dusenge [1,2,*], Sebastian González-Caro [2], Zorayda Restrepo [3] , Anna Gardner [2,4], Patrick Meir [5], Iain P. Hartley [2], Stephen Sitch [2], Adriana Sanchez [6], Juan Camilo Villegas [7], Lina M. Mercado [2,8,*]

[1] Division of Plant Sciences, Research School of Biology, The Australian National University, Canberra, ACT, Australia

[2] Faculty of Environment, Science, and Economy, University of Exeter, Exeter, United Kingdom

[3] Grupo de Servicios ecosistémicos y Cambio Climático, Corporación COL-TREE, Medellín, Colombia

[4] Birmingham Institute of Forest Research, University of Birmingham, Birmingham, United Kingdom

[5] School of Geosciences, University of Edinburgh, Edinburgh, United Kingdom

[6] Departamento de Biologia, Facultad de Ciencias Naturales, Universidad del Rosario, Bogotá, D.C., Colombia

[7] Grupo en Ecología Aplicada, Escuela Ambiental, Facultad de ingeniería, Universidad de Antioquia, Medellín, Colombia

[8] UK Centre for Ecology & Hydrology, Wallingford, United Kingdom

* Contact authors: Mirindi Eric Dusenge: MirindiEric.Dusenge@anu.edu.au; Lina M. Mercado: L.Mercado@exeter.ac.uk

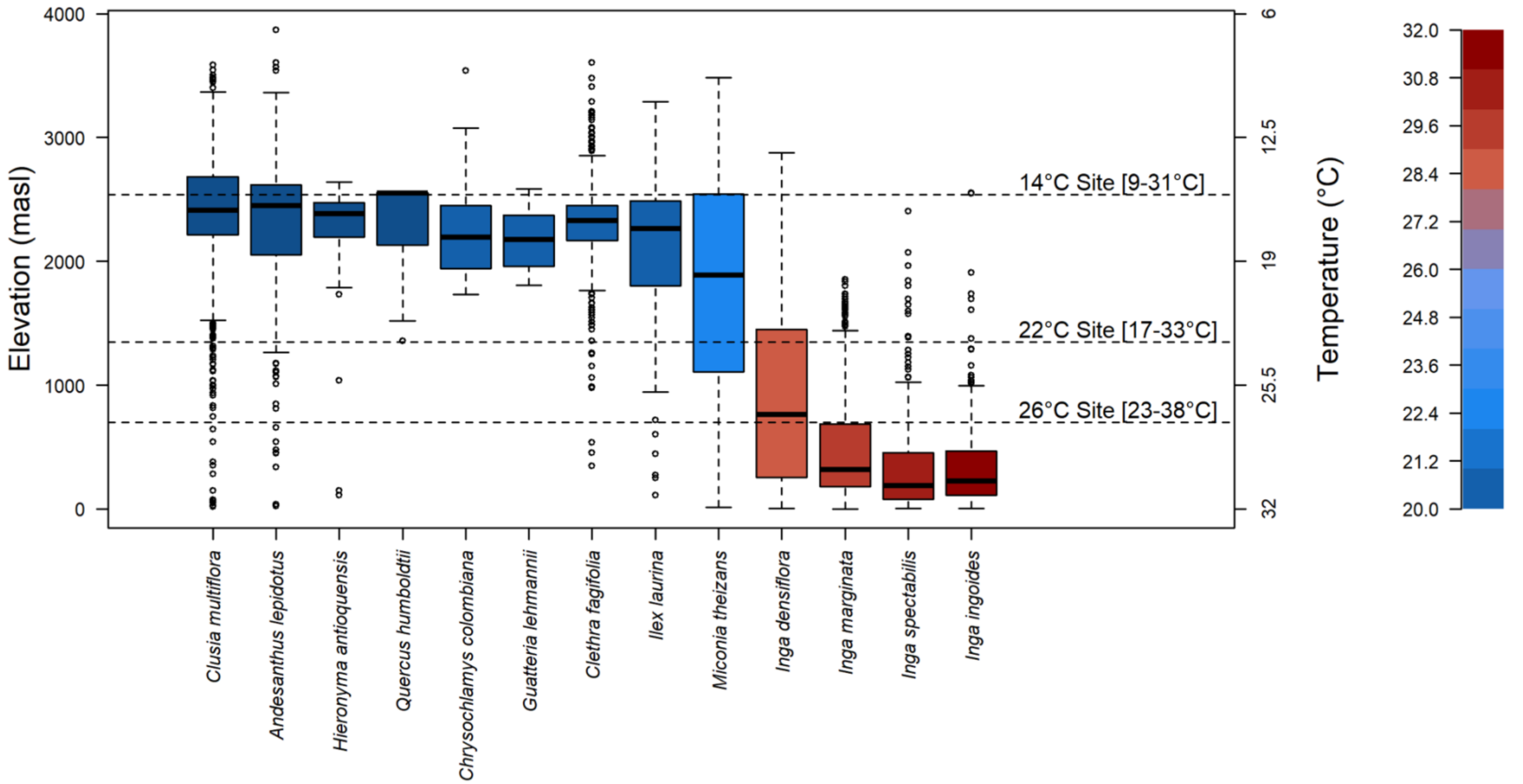

Supplementary Fig. 1 | Distribution of tree species in relation to their altitude and thermal niche of origin. Different color gradient represents species provenance (montane species = dark and light blue; lowland = dark and light red). Each box's upper and lower edges indicate the 75th and 25th percentiles, respectively, while the horizontal line is the median and vertical dashed lines indicate the 90th and 10th percentile ranges. Open circles represent outliers. Horizontal dashed lines represent each of the three site's mean growth temperature. The numbers on top of each line represent site mean annual temperature and numbers shown inside brackets represent the minima and maxima annual temperatures of each site. Color gradient bar on the right highlights approximately the average temperature of each species. The temperature data used to calculate species thermal distributions were extracted from the Worldclim database (Fick & Hijmans, 2017) at all the location where studied species have been recorded.

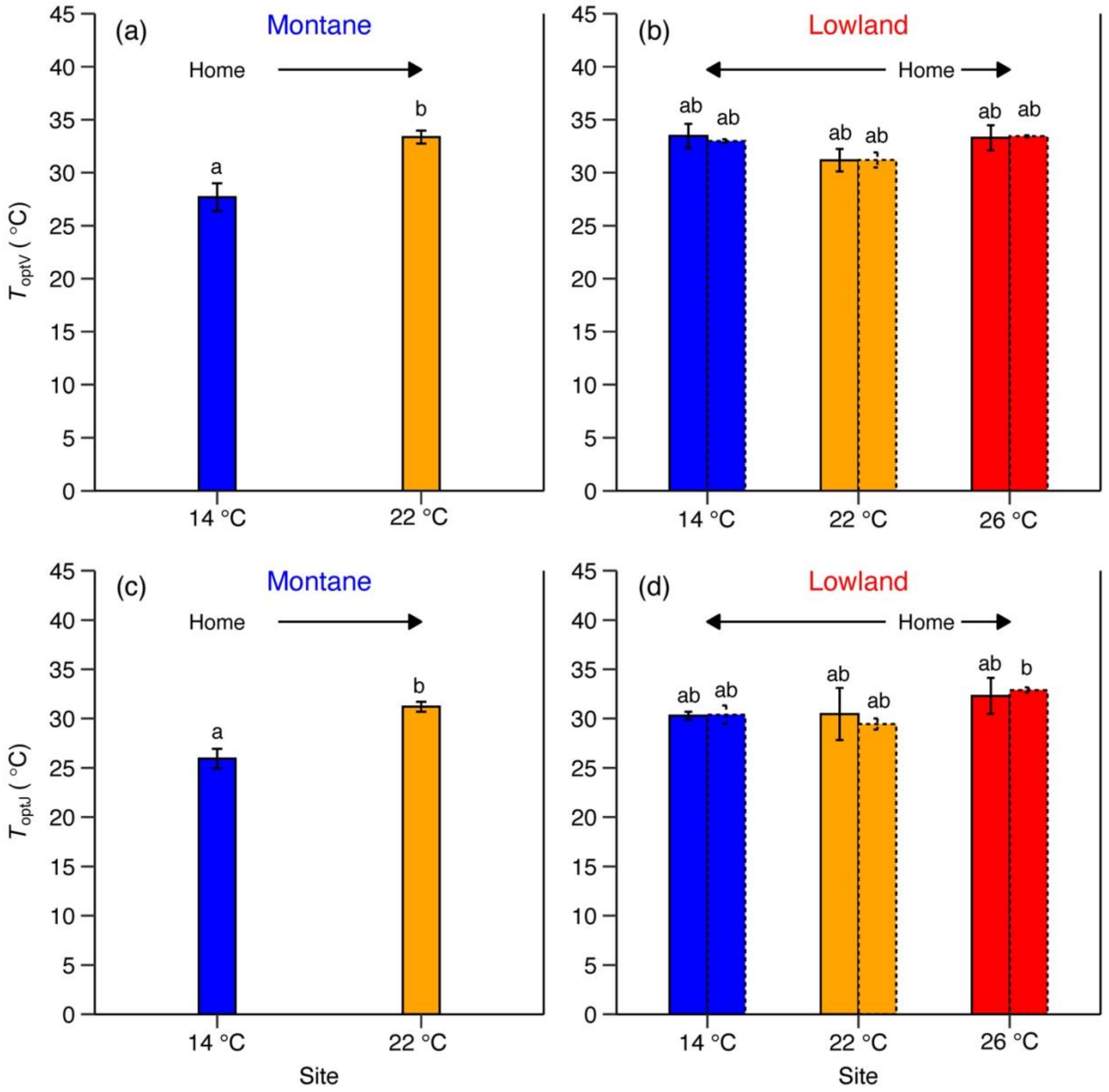

Supplementary Fig. 2 | Optima temperatures of underlying photosynthetic biochemical processes. Impact of growth temperature on the thermal optimum of the maximum Rubisco carboxylation rate (T_{optV}, °C) and the maximum electron transport rate (T_{optJ}, °C) in montane (a, c) and lowland (b, d) groups. The x-axis represents each site's mean annual temperature: 14 °C, 22 °C, and 26 °C (Supplementary Table 1). 'Home' indicates the native thermal environment for each species group (14 ° C for montane; 22 – 26 ° C for lowland), and arrows indicate if they were subjected to warming or cooling. In (b) and (d), the solid and dashed bars represent lowland species originating from the 22 °C and 26 °C sites, respectively. Small letters are used for statistical comparisons among each species group and site combination, where a different letter denotes a significant difference at the $p < 0.05$ threshold from *Sidak posthoc* test. Further details on statistical analyses for this figure can be found in Supplementary Table 3.

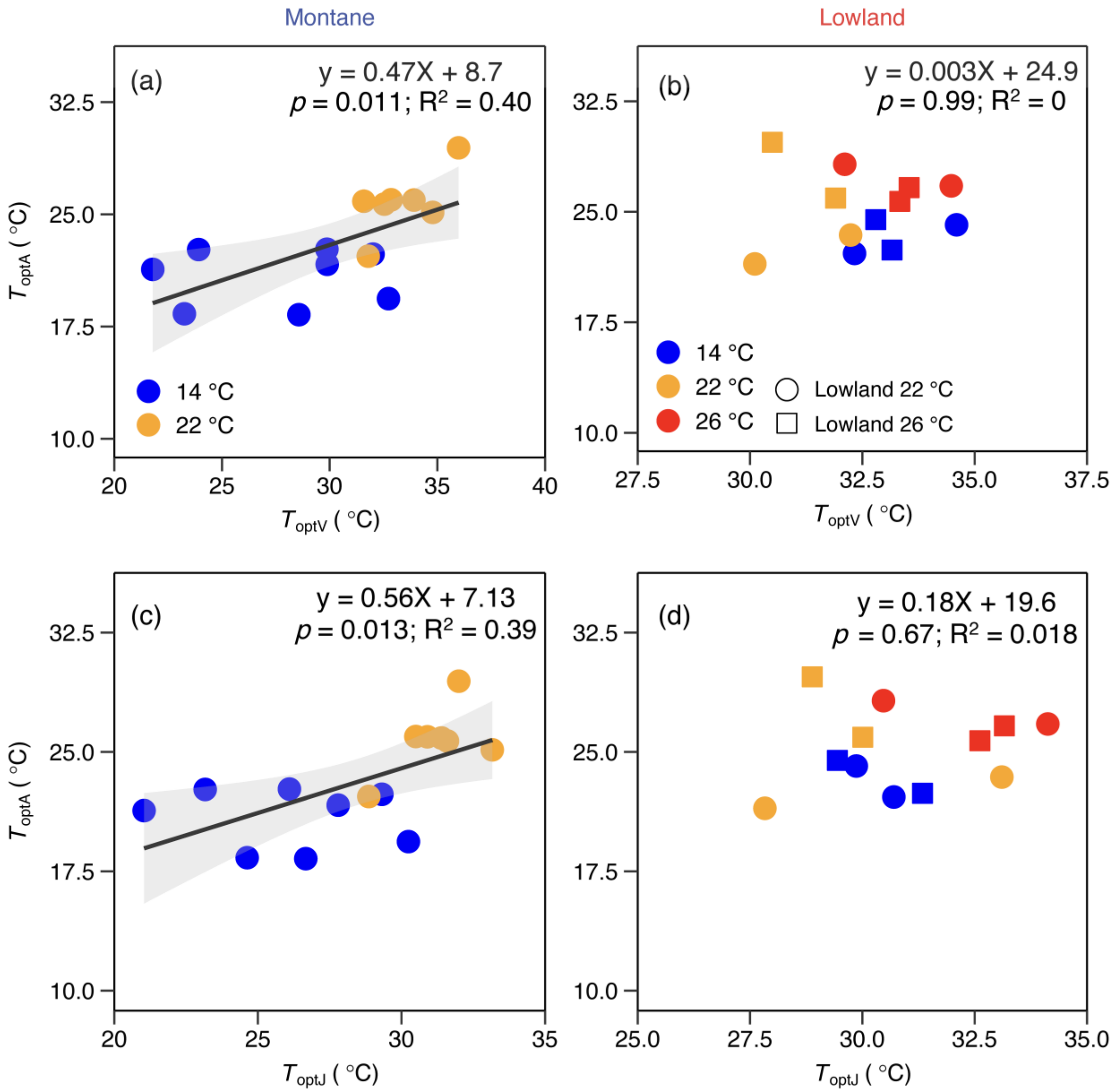

Supplementary Fig. 3 | Relationship between the optimum temperature of net photosynthesis and the optima temperatures of underlying biochemical processes. The temperature optimum of net photosynthesis measured at ambient CO_2 (T_{optA}, °C) as a function of the thermal optimum of the maximum Rubisco carboxylation rate (T_{optV}, °C) and the maximum electron transport rate (T_{optJ}, °C) in montane (a, c) and lowland (b, d) groups. Symbol colors represent mean annual temperature at experiment sites (14 °C = blue; 22 °C = orange; 26 °C = red). Symbol shapes in lowland group represent different provenances (circle = lowland species native at 22 °C site; square = lowland species native at 26 °C site). A simple linear regression model was used to analyse the relationship between these two variables across species and groups. The solid black line represents the regression line, and the light gray area represent 95 % confidence interval around the regression line.

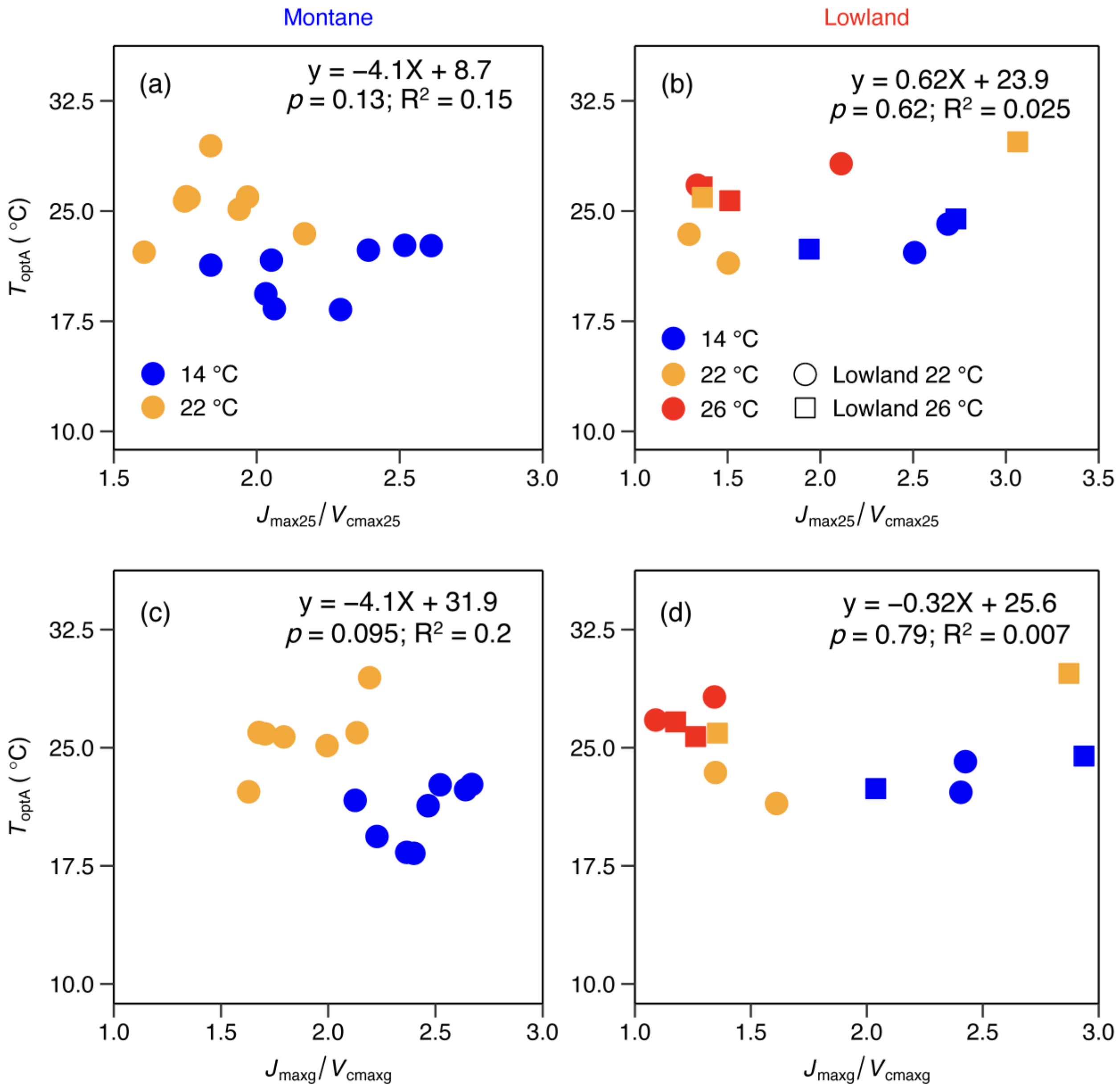

Supplementary Fig. 4 | Relationship between the optimum temperature of net photosynthesis and the optima temperatures of underlying biochemical processes. The temperature optimum of net photosynthesis measured at ambient CO_2 (T_{optA}, °C) as a function of the ratio of the maximum electron transport rate to Rubisco maximum carboxylation rate at a standard temperature of 25 °C (J_{max25}/V_{cmax25}), and the ratio of the maximum electron transport rate to Rubisco maximum carboxylation rate at the prevailing growth temperature (J_{maxg}/V_{cmaxg}) in montane (a, c) and lowland (b, d) groups. Symbol colors represent mean annual temperature at experiment sites (14 °C = blue; 22 °C = orange; 26 °C = red). Symbol shapes in lowland group represent different provenances (circle = lowland species native at 22 °C site; square = lowland species native at 26 °C site). A simple linear regression model was used to analyse the relationship between these two variables across species and groups.

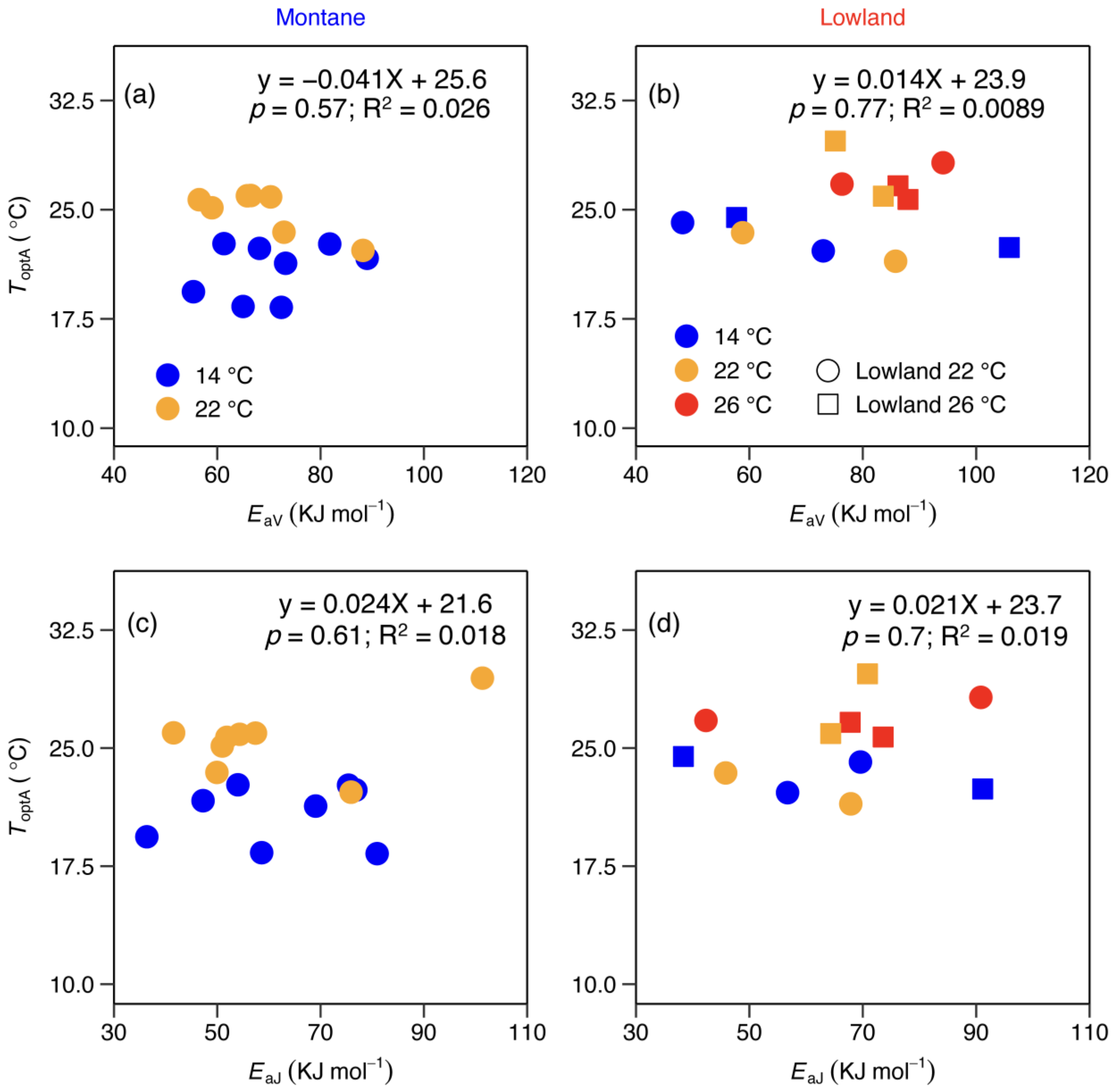

Supplementary Fig. 5 | The temperature optimum of net photosynthesis measured at ambient CO_2 (T_{optA}, °C) as a function of the activation energy of the maximum Rubisco carboxylation rate (E_{aV}, kJ mol^{-1}) amd the maximum electron transport rate (E_{aJ}, kJ mol^{-1}) in montane (a, c) and lowland (b, d) groups. Symbol colors represent mean annual temperature at experiment sites (14 °C = blue; 22 °C = orange; 26 °C = red). Symbol shapes in lowland group represent different provenances (circle = lowland species native at 22 °C site; square = lowland species native at 26 °C site). A simple linear regression model was used to analyse the relationship between these two variables across species and groups.

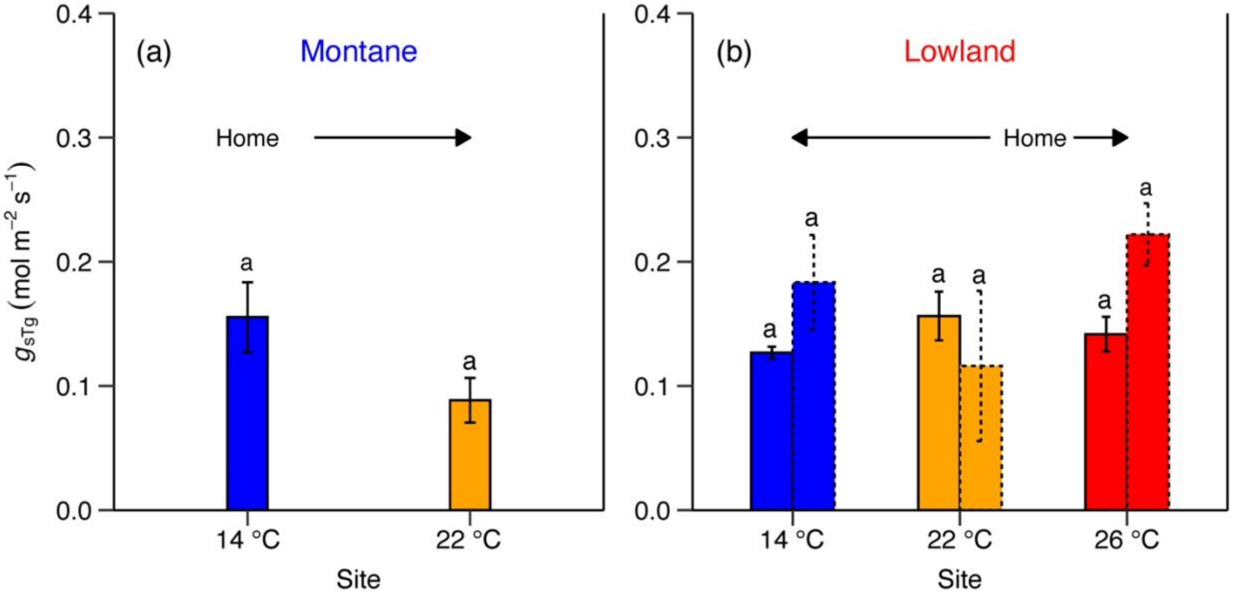

Supplementary Fig. 6 | Stomatal conductance. Impact of growth temperature on stomatal conductance measured at prevailing growth temperature (g_{sTg}, mol m^{-2} s^{-1}) in (a) montane and (b) lowland tree species. The x-axis represents each site's mean annual temperature: 14 °C, 22 °C, and 26 °C (Supplementary Table 1). g_{sTg} was retrieved from the $A - C_i$ curves at leaf temperature of 20 °C, 25 °C, and 30 °C for the 14 °C, 22 °C and 26 °C sites, respectively, and these leaf temperatures were approximately close to mean daytime prevailing growth temperatures of 21.4 °C, 24.3 °C and 31.2 °C, respectively. 'Home' indicates the native thermal environment for each species group (14 ° C for montane; 22 – 26 ° C for lowland), and arrows indicate if they were subjected to warming or cooling. In (b), the solid and dashed bars represent lowland species originating from the 22 °C and 26 °C sites, respectively. Small letters are used for statistical comparisons among each species group and site combination, where a different letter denotes a significant difference at the $p < 0.05$ threshold from *Sidak posthoc* test. Further details on statistical analyses for this figure can be found in Supplementary Table 3.

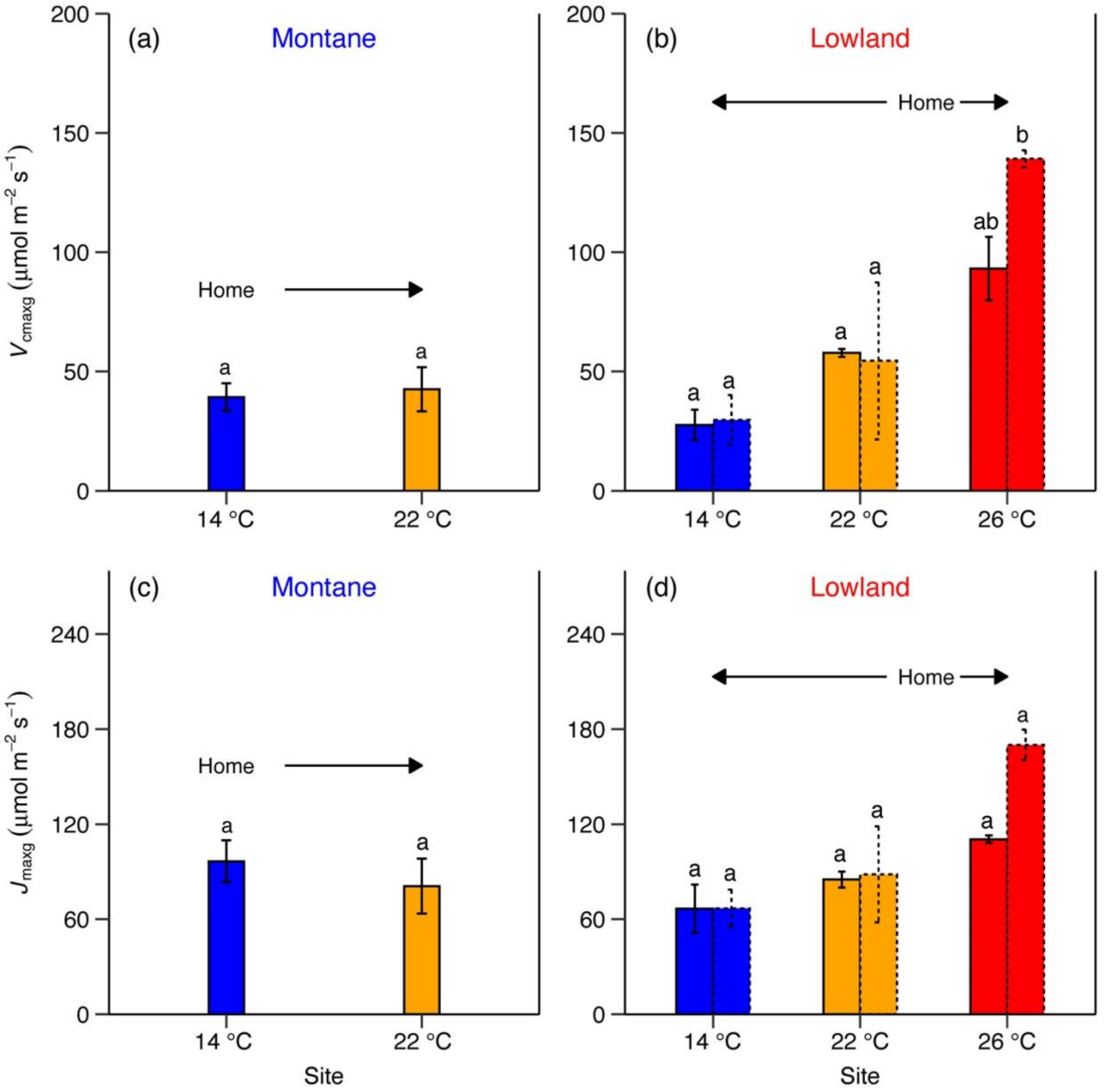

Supplementary Fig. 7 | Photosynthetic capacity at growth temperature. Impact growth temperature on photosynthetic capacity estimated at prevailing growth temperature in montane (a, c) and lowland (c, d) groups. (a, b) The maximum carboxylation rate of Rubisco at growth temperature (V_{cmaxg}; µmol m^{-2} s^{-1}) and (c, d) the maximum electron transport rate at growth temperature (J_{maxg}; µmol m^{-2} s^{-1}). The x-axis represents each site's mean annual temperature: 14 °C, 22 °C, and 26 °C (Supplementary Table 1). However, estimation of rates of V_{cmaxg} and J_{maxg} was done using mean daytime (6 am – 6 pm) growth temperature (14 °C site = 21.4 °C, 22 °C site = 24.3 °C, and 26 °C = 31.2 °C) of one month prior to each measurement campaign at each site, the time defined as the thermal acclimation period of photosynthesis 'Home' indicates the native thermal environment for each species group (14 ° C for montane; 22 – 26 ° C for lowland), and arrows indicate if they were subjected to warming or cooling. In (b), the solid and dashed bars represent lowland species originating from the 22 °C and 26 °C sites, respectively. Small letters

are used for statistical comparisons among each species group and site combination, where a different letter denotes a significant difference at the $p < 0.05$ threshold from *Sidak posthoc* test. Further details on statistical analyses for this figure can be found in Supplementary Table 3.

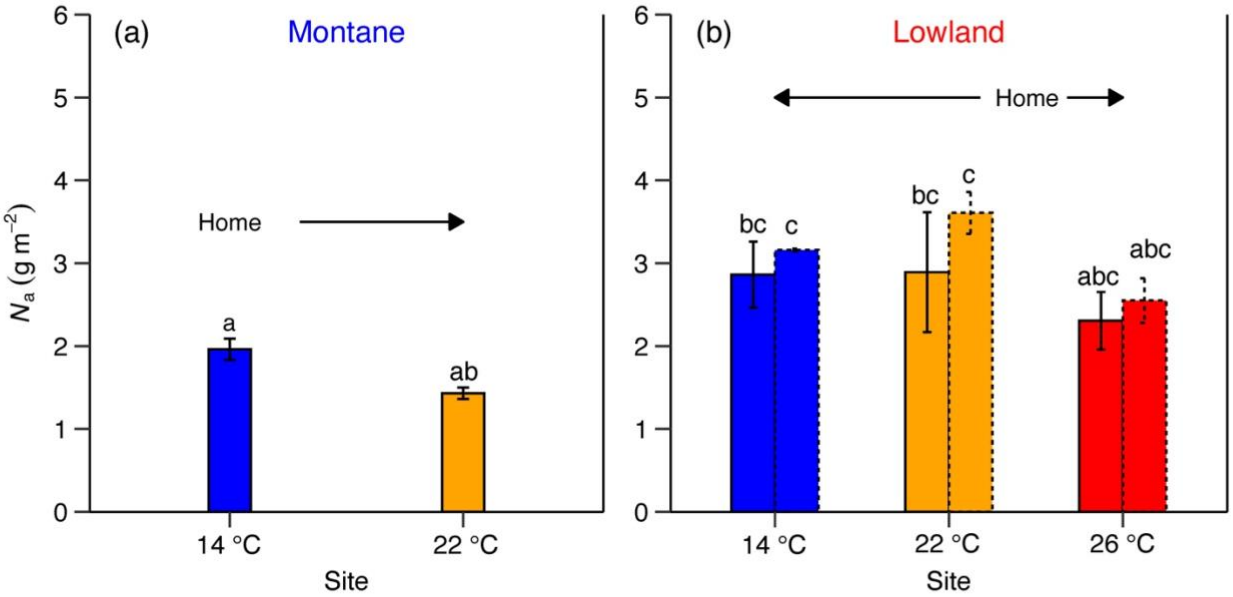

Supplementary Fig. 8 | Leaf nitrogen concentration. Impact of growth temperature on leaf nitrogen concentration per unit leaf area (N_a; g m^{-2}) in montane (a) and lowland (b) groups. The x-axis represents each site's mean annual temperature: 14 °C, 22 °C, and 26 °C (Supplementary Table 1). 'Home' indicates the native thermal environment for each species group (14 ° C for montane; 22 – 26 ° C for lowland), and arrows indicate if they were subjected to warming or cooling. In (b), the solid and dashed bars represent lowland species originating from the 22 °C and 26 °C sites, respectively. Small letters are used for statistical comparisons among each species group and site combination, where a different letter denotes a significant difference at the $p < 0.05$ threshold from *Sidak posthoc* test. Further details on statistical analyses for this figure can be found in Supplementary Table 3.

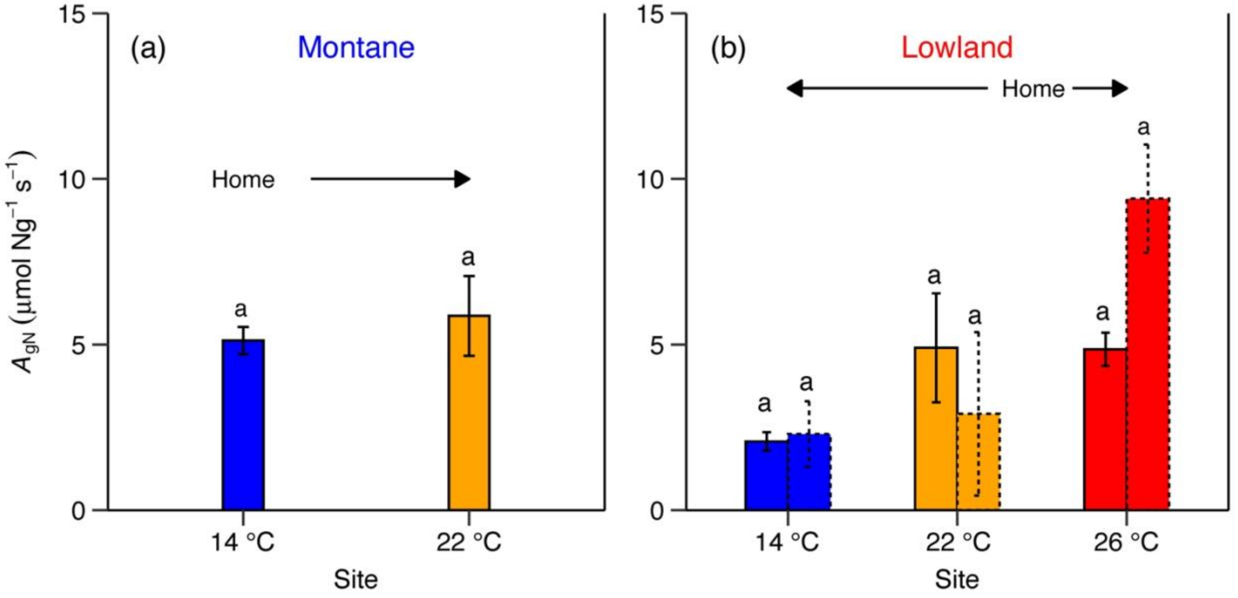

Supplementary Fig. 9 | Normalized rates of net photosynthesis to leaf nitrogen at growth temperature. The impact of growth temperature on the normalized rates of net photosynthesis to leaf nitrogen concentration at ambient CO_2 and prevailing growth temperature (A_{gN}, μmol Ng^{-2} s^{-1}) in (a) montane and (b) lowland tree species. The x-axis represents each site's mean annual temperature: 14 °C, 22 °C, and 26 °C (Supplementary Table 1). However, estimation of A_g rates was done using mean daytime (6 am – 6 pm) growth temperature (14 °C site = 21.4 °C, 22 °C site = 24.3 °C, and 26 °C = 31.2 °C) of one month prior to each measurement campaign at each site, the time defined as the thermal acclimation period of photosynthesis. 'Home' indicates the native thermal environment for each species group (14 ° C for montane; 22 – 26 ° C for lowland), and arrows indicate if they were subjected to warming or cooling. In (b), the solid and dashed bars represent lowland species originating from the 22 °C and 26 °C sites, respectively. Small letters are used for statistical comparisons among each species group and site combination, where a different letter denotes a significant difference at the $p < 0.05$ threshold from *Sidak posthoc* test. Further details on statistical analyses for this figure can be found in Supplementary Table 3.

Supplementary Table 1 | Climate data of the experimental sites

	26°C site	22°C	14°C
	Low elevation	Mid elevation	High elevation
Latitude & Longitude	6.844ºN &-75.810ºW	5.541ºN & -75.685ºW	5.513ºN & -75.678ºW
Elevation (masl)	736	1357	2516
MAT (℃)	25.58	22.14	13.78
MAP (mm yr^{-1})	2298	2045	2774
T_{day} (℃)	27.1	22.14	16.1
T_{night} (℃)	22.7	19.6	12.6
VPD_{day} (kPa)	1.83	1.14	0.82
Direct PAR mean	NA	682	580
Diffuse PAR mean	NA	332	368
Maximum number of consecutive days with no rain	20.3	21.4	12.5
Soil properties			
Phosphorus P ($Kgha^{-1}$)	13.95	29.15	10.5
Nitrogen N ($Kgha^{-1}$)	201	204.5	231
pH	5.3	5.3	5.1

The weather data are mean values for the period of October 1st 2019 until January 31st 2022. Mean daytime (T_{day}) and night-time (T_{night}) temperatures were calculated from 06:00–17:59 and 18:00–05:59 respectively. Mean daytime vapour pressure deficit (VPD_{day}) was calculated using 06:00–17:59 values. Native soil data at experimental sites represent the mean of the top 0–30 cm taken from three random samples at each experimental site.

Supplementary Table 2 | Environmental data of the study sites for one month prior to gas exchange measurements

	26°C site	22°C	14°C
	Low elevation	Mid elevation	High elevation
T_{day}	31.16	24.25	21.38
T_{night}	19.11	19.28	12.09
T_{min}	23.62	17.39	9.04
T_{max}	38.53	33.48	31.22
VPD_{mean}	1.85	1.39	0.87
VPD_{max}	3.89	2.78	2.37

Mean annual temperature (MAT, °C); daytime temperature (6 am to 6 pm; T_{day}, °C); nighttime temperature (6 pm to 6 am, T_{night}, °C); minimum temperature (T_{min}, °C); maximum temperature (T_{max}, °C); mean vapor pressure deficit (VPD_{mean}, kPa); maximum vapor pressure deficit (VPD_{max}, kPa).

Supplementary Table 3 | Thermal sensitivity parameters of net photosynthesis of the studied species across different sites

Species	Provenance	Site	n	T_{optA}	T_{max}	*b*
Andesanthus lepidota	Montane	14 °C	2	18.29 ± 3.20	40.28 ± 0.48	0.05 ± 0.02
		22 °C	1	22.20 ± NA	40.54 ± NA	0.06 ± NA
Clethra fagifolia	Montane	14 °C	4	22.34 ± 1.84	41.97 ± 2.11	0.03 ± 0
		22 °C	3	25.13 ± 0.22	44.32 ± 0.88	0.02 ± 0
Clusia multiflora	Montane	14 °C	3	21.32 ± 0.69	35.89 ± 0.86	0.06 ± 0.01
		22 °C	1	29.44 ± NA	40.53 ± NA	0.06 ± NA
Guatteria lehmannii	Montane	14 °C	1	22.67 ± NA	36.48 ± NA	0.05 ± NA
		22 °C	3	25.88 ± 1.43	39.91 ± 0.58	0.02 ± 0.01
Hieronyma antioquensis	Montane	14 °C	1	22.64 ± NA	35.34 ± NA	0.06 ± NA
		22 °C	1	23.45 ± NA	39.52 ± NA	0.04 ± NA
Ilex laurina	Montane	14 °C	3	18.35 ± 2.62	39.13 ± 2.94	0.02 ± 0.01
		22 °C	3	25.68 ± 1.04	40.49 ± 1.94	0.02 ± 0
Inga densiflora	Lowland 22	14 °C	2	22.17 ± 0.65	41.48 ± 2.36	0.02 ± 0
		26 °C	4	26.76 ± 0.75	43.06 ± 0.85	0.05 ± 0.01
		22 °C	2	23.42 ± 1.04	40.64 ± 0.17	0.04 ± 0.01
Inga ingoides	Lowland 26	14 °C	3	22.40 ± 3.04	40.16 ± 0.57	0.04 ± 0.01
		26 °C	3	26.64 ± 0.78	42.35 ± 0.97	0.07 ± 0.01
		22 °C	4	25.93 ± 0.45	41.31 ± 0.44	0.07 ± 0.01
Inga marginata	Lowland 22	14 °C	3	24.11 ± 1.28	38.95 ± 1.25	0.03 ± 0.01
		26 °C	4	28.22 ± 0.91	40.95 ± 0.33	0.07 ± 0.02
		22 °C	3	21.46 ± 1.67	40.62 ± 1.08	0.03 ± 0.01
Inga spectabilis	Lowland 26	14 °C	2	24.47 ± 2.18	40.22 ± 2.58	0.02 ± 0.01
		26 °C	3	25.71 ± 1.88	42.17 ± 1.26	0.09 ± 0.03
		22 °C	2	29.72 ± 0.58	40.21 ± 0.73	0.02 ± 0
Miconia theizans	Montane	14 °C	2	19.37 ± 0.09	43.51 ± 0.30	0.02 ± 0
		22 °C	3	25.95 ± 1.58	44.55 ± 1.77	0.03 ± 0.01
Quercus humboldtii	Montane	14 °C	2	21.66 ± 2.14	38.07 ± 3.16	0.02 ± 0.01
		22 °C	3	25.97 ± 1.18	41.90 ± 0.55	0.03 ± 0.01

Thermal optimum of net photosynthesis (T_{optA}, °C); High-temperature CO_2 compensation point (T_{max}; °C); the breadth of the temperature response curve of net photosynthesis (*b*, unitless)

Supplementary Table 4 | Summary report of ANOVA showing degrees of freedom, F-values, p-values on photosynthetic traits

		DF	DF_{res}	*F*-value	*P*-value
T_{optV}	Site	2	20	5.5	**0.012**
	Provenance	2	20	1.2	0.3
	Site:Provenance	3	20	3.5	**0.033**
T_{optJ}	Site	2	20	11.4	**0.00049**
	Provenance	2	20	1.5	0.25
	Site: Provenance	3	20	2.8	*0.066*
E_{aV}	Site	2	21	2.3	0.12
	Provenance	2	21	1.1	0.3
	Site: Provenance	3	21	0.4	0.7
E_{aJ}	Site	2	21	0.3	0.8
	Provenance	2	21	0.2	0.8
	Site: Provenance	3	21	0.047	0.9
g_{sTg}	Site	2	20	2.9	0.072
	Provenance	2	20	0.7	0.5
	Site: Provenance	3	20	0.9	0.5
V_{cmaxg}	Site	2	20	10.1	**<0.0001**
	Provenance	2	20	0.8	0.5
	Site: Provenance	3	20	1.7	0.2
J_{maxg}	Site	2	20	3.7	**0.043**
	Provenance	2	20	0.78	0.47
	Site: Provenance	3	20	0.9	0.4
N_a	Site	2	21	1.9	0.17
	Provenance	2	21	36.5	**<0.0001**
	Site: Provenance	3	21	1.9	0.15
A_{gN}	Site	2	20	2.3	0.12
	Provenance	2	20	2.9	0.074
	Site: Provenance	3	20	1.3	0.29
J_{max25}/V_{cmax25}	Site	2	21	8.5	**0.002**
	Provenance	2	21	0.18	0.83

	Site: Provenance	3	21	1.9	0.15
J_{maxg}/V_{cmaxg}	Site	2	20	22.3	**<0.0001**
	Provencance	2	20	0.74	0.49
	Site:Provenance	3	20	0.64	0.6

Maximum Rubisco carboxylation rate (T_{optV}, °C); maximum electron transport rate (T_{optJ}, °C); activation energy of the maximum Rubisco carboxylation rate (E_{aV}, kJ mol^{-1}); c, d) activation energy of the maximum electron transport rate (E_{aJ}, kJ mol^{-1}); stomatal conductance at prevailing growth temperature (g_{sTg}, mol m^{-2} s^{-1}); maximum carboxylation rate of Rubisco at growth temperature at growth temperature (V_{cmaxg}; μmol m^{-2} s^{-1}); maximum electron transport rate at growth temperature (J_{maxg}; μmol m^{-2} s^{-1}); leaf nitrogen concentration per unit leaf area (N_a; g m^{-2}); normalized rates of net photosynthesis to leaf nitrogen concentration at ambient CO_2 and prevailing growth temperature (A_{gN}, μmol Ng^{-2} s^{-1}); ratio of the maximum electron transport rate to Rubisco maximum carboxylation rate at a standard temperature of 25 °C (J_{max25}/V_{cmax25}); ratio of the maximum electron transport rate to Rubisco maximum carboxylation rate at the prevailing growth temperature (J_{maxg}/V_{cmaxg}).

Supplementary Table 5 | Thermal sensitivity parameters of the photosynthetic biochemical processes of the studied species across different sites

Species	Provenance	Site	n	T_{optV}	T_{optJ}	Ea_{Vcmax}	Ea_{Jmax}
		14 °C	4	28.57±0.54	26.67±1.20	72.39±13.88	80.95±11.84
Andesanthus lepidota	Montane	22 °C	1	31.79±NA	28.86±NA	88.21±NA	75.90±NA
Chrysochlamys colombiana	Montane	14 °C	1	27.16±NA	24.45±NA	60.02±NA	41.23±NA
		14 °C	3	32.01±2.13	29.32±0.93	68.17±8.09	76.83±30.58
Clethra fagifolia	Montane	22 °C	4	34.78±0.2	33.16±0.55	58.96±3.46	50.97±3.49
		14 °C	1	21.79±NA	21.04±NA	73.23±NA	69.04±NA
Clusia multiflora	Montane	22 °C	1	35.98±NA	32.00±NA		101.33±NA
		14 °C	3	29.87±2.11	26.10±1.18	61.30±14.74	53.99±6.21
Guatteria lehmannii	Montane	22 °C	4	31.59±1.16	31.39±1.52	70.33±6.36	54.34±13.36
		14 °C	4	23.92±0.7	23.16±0.59	81.77±5.83	75.41±4.12
Hieronyma antioquensis	Montane	22 °C		NA	NA	72.91±12.66	49.93±21.89
		14 °C	1	23.25±NA	24.63±2.74	64.99±21.21	58.59±20.83
Ilex laurina	Montane	22 °C	4	32.54±1.7	31.60±2.12	56.51±9.66	51.86±2.84
		14 °C	3	32.33±1.26	30.70±1.15	73.03±16.12	56.74±10.17
		26 °C	4	34.48±0.39	34.13±0.56	76.33±4.84	42.34±5.27
Inga densiflora	Lowland 22	22 °C	2	32.24±1.8	33.10±1.31	58.81±15.31	45.82±6.9
		14 °C	4	33.16±0.83	31.34±0.68	105.85±21.74	91.14±17.14
		26 °C	3	33.54±0.86	33.16±1.22	86.21±2.97	67.78±6.16
Inga ingoides	Lowland 26	22 °C	4	31.90±0.42	30.01±0.35	83.62±3.55	64.34±3.30
Inga marginata	Lowland 22	14 °C	3	34.60±1.02	29.87±1.24	48.22±4.5	69.59±26.09

		26 °C	4	32.11±1.12	30.47±0.59	94.16±8	90.82±16.16
		22 °C	4	30.11±1.06	27.83±0.87	85.79±8.55	67.88±8.92
		14 °C	4	32.80±0.26	29.44±0.76	57.74±6.43	38.37±5.28
		26 °C	3	33.33±1.21	32.62±2.65	87.97±12.79	73.60±17.75
Inga spectabilis	Lowland 26	22 °C	3	30.50±1.37	28.88±0.63	75.16±17.28	70.84±2.41
		14 °C	4	32.73±1.1	30.24±1.07	55.39±8.25	36.36±9.67
Miconia theizans	Montane	22 °C	3	33.91±1.43	30.90±1.58	65.83±4.49	57.40±2.25
		14 °C	2	29.88±3.65	27.80±3.2	89.02±6.37	47.24±13.42
Quercus humboldtii	Montane	22 °C	3	32.85±2.36	30.50±2.73	66.48±5.6	41.54±14.44

Thermal optimum of the maximum Rubisco carboxylation rate (T_{optV}, °C); maximum electron transport rate (T_{optJ}, °C); activation energy of the maximum Rubisco carboxylation rate (E_{aV}, kJ mol^{-1}); maximum electron transport rate (E_{aJ}, kJ mol^{-1})

Supplementary Table 6 | Summary report of unpaired t-tests.

**Provenance**	T_{opt}	***t*-value**	**DF**	***p*-value**
Montane	T_{opt287}	-1.2	28	0.2
Lowland 22 °C	T_{opt287}	-0.8	10	0.4
Lowland 26 °C	T_{opt287}	-0.07	10	0.9
Montane	$T_{optgross}$	1.1	24	0.3
Lowland 22 °C	$T_{optgross}$	0.008	10	0.9
Lowland 26 °C	$T_{optgross}$	0.2	10	0.9

T-tests between thermal optimum of net photosynthesis at measured intercellular CO_2 concentration (C_i, µmol m^{-2}) - T_{optA} and the thermal optimum of net photosynthesis at a common C_i of 287 µmol m^{-2} - T_{opt287}; and between thermal optimum of net photosynthesis at measured C_i - T_{optA} and the thermal optimum of gross photosynthesis - $T_{optgross}$. We assumed equal variance, and this condition was met for all tests.

Supplementary Table 7 | Rates of net photosynthesis and underlying biochemical processes at the prevailing growth temperature

**Species**	**Provenance**	**Site**	**n (A_g)**	A_{growth}	**n**	V_{cmaxg}	J_{maxg}	J_{maxg}/V_{cmaxg}
		14 °C	2	19.92±1.85	4	71.70±11.81	166.68±20.22	2.40±0.23
Andesanthus lepidota	Montane	22 °C	1	20.28±NA	2	93.81±14.37	176.21±NA	1.63±NA
Chrysochlamys colombiana	Montane	14 °C		NA	1	32.37±NA	98.63±NA	3.05±NA
		14 °C	4	9.72±1.51	3	34.37±8.88	86.66±13.93	2.64±0.25
Clethra fagifolia	Montane	22 °C	3	8.33±1.99	4	34.67±7.19	66.66±12.72	1.99±0.18
		14 °C	3	12.31±1.15	1	38.80±NA	95.67±NA	2.47±NA
Clusia multiflora	Montane	22 °C	1	5.77±NA	1	25.56±NA	56.04±NA	2.19±NA
		14 °C	1	8.95±NA	3	31.32±3.42	83.61±9.54	2.67±0.06
Guatteria lehmannii	Montane	22 °C	3	3.83±0.89	4	20.13±4.18	34.02±6.56	1.71±0.09

Hieronyma antioquensis	Montane	14 °C	1	9.77±NA	4	55.36±4.91	138.27±8.10	2.52±0.10
		22 °C	1	11.47±NA		NA	NA	NA
Ilex laurina	Montane	14 °C	3	6.70±2.06	1	13.94±NA	32.98±NA	2.37±NA
		22 °C	3	4.98±1.59	4	36.19±7.10	61.67±7.51	1.79±0.18
Inga densiflora	Lowland 22	14 °C	2	7.68±2.28	3	34.01±6.93	81.88±16.56	2.41±0.11
		26 °C	4	11.76±1.80	4	106.45±25.98	112.96±24.35	1.09±0.08
		22 °C	2	11.25±3.90	2	59.40±22.01	80.00±29.53	1.35±0
Inga ingoides	Lowland 26	14 °C	3	10.71±1.16	4	40.11±6.31	78.73±5.94	2.04±0.18
		26 °C	3	16.17±1.91	3	135.63±17.50	160.31±24.43	1.18±0.03
		22 °C	4	16.98±0.92	4	87.29±6.78	118.60±12.69	1.36±0.09
Inga marginata	Lowland 22	14 °C	3	4.61±0.22	3	21.28±0.79	51.38±2.84	2.43±0.19
		26 °C	4	10.63±2.57	4	79.73±20.22	108.04±28.01	1.34±0.08
		22 °C	3	10.88±2	3	56.06±12.55	90.13±20.34	1.61±0.05
Inga spectabilis	Lowland 26	14 °C	2	4.02±2.21	4	19.33±3.33	54.99±8.88	2.94±0.27
		26 °C	3	18.26±2.65	3	142.71±14.81	179.69±18.21	1.26±0.04
		22 °C	2	1.75±0.7	3	21.61±5.91	58.01±9.48	2.87±0.36
Miconia theizans	Montane	14 °C	2	13.97±0.36	4	49.50±7.68	109.43±16.59	2.23±0.10
		22 °C	3	9.95±1.5	3	45.13±7.32	95.53±13.99	2.13±0.08
Quercus humboldtii	Montane	14 °C	2	5.76±1.39	2	26.63±6.11	57.12±15.17	2.13±0.08
		22 °C	3	8.66±2.94	3	42.27±10.68	76.24±31.67	1.68±0.27

Rates of net photosynthesis estimated at growth temperature conditions (A_{growth}, µmol m^{-2} s^{-1}); maximum carboxylation rate of Rubisco at growth temperature at growth temperature (V_{cmaxg}; µmol m^{-2} s^{-1}); maximum electron transport rate at growth temperature (J_{maxg}; µmol m^{-2} s^{-1}); ratio of J_{maxg} to V_{cmaxg}.

Supplementary Table 8 | Rates of photosynthetic capacity at a common temperature of 25 °C and leaf nitrogen concentration on an area basis

Species	Provenance	Site	n	V_{cmax25}	J_{max25}	J_{max25}/V_{cmax25} ratio	n (N_a)	N_a
		14 °C	4	87.35±14.09	195±23.71	2.29±0.19	3	2.55±0.06
Andesanthus lepidota	Montane	22 °C	2	90.11±13.66	166.67±NA	1.61±NA	2	1.59±0.04
Chrysochlamys colombiana	Montane	14 °C	1	38.07±NA	104.19±NA	2.74±NA	1	1.81±NA
		14 °C	4	46.37±7.25	108.47±11.77	2.39±0.11	2	1.75±0.25
Clethra fagifolia	Montane	22 °C	4	35.29±7.91	66.16±13.05	1.94±0.14	4	1.56±0.07
		14 °C	3	60.39±4.28	109.47±5.52	1.84±0.2	3	2.44±0.31
Clusia multiflora	Montane	22 °C	3	19.67±2.11	35.27±5.72	1.84±0.34	2	1.60±0.20
		14 °C	3	36.56±7.05	90.36±15.13	2.52±0.17	2	2.23±0.54
Guatteria lehmannii	Montane	22 °C	4	20.02±4.65	34.35±6.92	1.76±0.17	3	1.44±0.10
		14 °C	4	61.21±4.52	158.76±7.85	2.61±0.08	3	1.60±0.33
Hieronyma antioquensis	Montane	22 °C	2	47.06±12.42	95.68±3.07	2.17±0.51	2	1.64±0.07
		14 °C	4	39.44±6.84	82.45±16.34	2.06±0.09	3	1.77±0.34
Ilex laurina	Montane	22 °C	4	38.31±8.40	62.19±7.85	1.75±0.24	3	1.27±0.24
		14 °C	3	42.31±9.21	104.88±21.97	2.51±0.32	2	3.26±0.96
		26 °C	4	65.60±15.26	86.33±18.27	1.34±0.04	3	2.65±0.73
Inga densiflora	Lowland 22	22 °C	3	56.48±11.87	72.56±14.13	1.29±0.02	2	3.61±0.37
		14 °C	4	56.57±8.23	103.46±7.71	1.94±0.29	4	3.14±0.24
		26 °C	3	78.44±7.26	106.68±8.79	1.36±0.02	3	2.28±0.55
Inga ingoides	Lowland 26	22 °C	4	75.33±6.60	102.97±11.35	1.36±0.08	3	3.36±0.32
		14 °C	4	25.66±2.64	66.39±2.67	2.69±0.34	4	2.46±0.19
		26 °C	4	45.61±10.83	87.17±17.52	2.11±0.37	4	1.96±0.31
Inga marginata	Lowland 22	22 °C	3	55.55±12.25	84.79±20.94	1.50±0.05	3	2.17±0.33
		14 °C	4	21.27±3.60	55.70±11.81	2.73±0.44	4	3.18±0.32
		26 °C	3	90.65±11.62	138.67±23.48	1.51±0.08	3	2.82±0.95
Inga spectabilis	Lowland 26	22 °C	3	19.72±6.02	55.25±9.57	3.06±0.44	2	3.86±0.33
		14 °C	4	62.83±13.94	126.62±27.25	2.03±0.08	4	2.09±0.24
Miconia theizans	Montane	22 °C	4	37.76±7.50	75.88±16.81	1.97±0.13	4	1.21±0.31
		14 °C	3	31.49±6.30	63.88±13.49	2.05±0.18	4	1.42±0.17
Quercus humboldtii	Montane	22 °C	4	37.40±8.03	68.62±22.50	1.75±0.21	2	1.15±0.05

Maximum carboxylation rate of Rubisco at growth temperature at a common temperature of 25 °C (V_{cmax25}; µmol m^{-2} s^{-1}); maximum electron transport rate at a common temperature of 25 °C (J_{max25}; µmol m^{-2} s^{-1}); ratio of J_{max25} to V_{cmax25}; nitrogen concentration on area basis (N_a, g m^{-2})
